# Genome-scale target identification in *Escherichia coli* for high-titer production of free fatty acids

**DOI:** 10.1038/s41467-021-25243-w

**Published:** 2021-08-17

**Authors:** Lixia Fang, Jie Fan, Shulei Luo, Yaru Chen, Congya Wang, Yingxiu Cao, Hao Song

**Affiliations:** 1 Frontier Science Center for Synthetic Biology and Key Laboratory of Systems Bioengineering (Ministry of Education), Tianjin University, Tianjin, China; 2 Collaborative Innovation Center of Chemical Science and Engineering (Tianjin), School of Chemical Engineering and Technology, Tianjin University, Tianjin, China

**Keywords:** High-throughput screening, Metabolic engineering, Applied microbiology, Synthetic biology

## Abstract

To construct a superior microbial cell factory for chemical synthesis, a major challenge is to fully exploit cellular potential by identifying and engineering beneficial gene targets in sophisticated metabolic networks. Here, we take advantage of CRISPR interference (CRISPRi) and omics analyses to systematically identify beneficial genes that can be engineered to promote free fatty acids (FFAs) production in *Escherichia coli*. CRISPRi-mediated genetic perturbation enables the identification of 30 beneficial genes from 108 targets related to FFA metabolism. Then, omics analyses of the FFAs-overproducing strains and a control strain enable the identification of another 26 beneficial genes that are seemingly irrelevant to FFA metabolism. Combinatorial perturbation of four beneficial genes involving cellular stress responses results in a recombinant strain *ihfA*^L−^-*aidB*^+^-*ryfA*^M−^-*gadA*^H−^, producing 30.0 g L^−1^ FFAs in fed-batch fermentation, the maximum titer in *E. coli* reported to date. Our findings are of help in rewiring cellular metabolism and interwoven intracellular processes to facilitate high-titer production of biochemicals.

## Abstract (web summary)

Identification of gene targets is one of the major challenges to construct superior microbial cell factory for chemical synthesis. Here, the authors employ CRISPRi and omics analyses for genome-scale target genes identification for high-titer production of free fatty acids in *E. coli*.

## Introduction

Microbial biosynthesis of a desired product is a sophisticated process that usually involves the coordination of multiple metabolic intermediates and reactions that are subject to complex interactions^1,2^. On the one hand, each of these intermediates flows to other metabolic pathways for the biosynthesis of cellular components or metabolites, acting as one of the multibranched nodes in the topology of metabolic landscapes^3,4^. On the other hand, each reaction in the biosynthetic pathway relies greatly on many cellular processes, such as gene transcription and translation to enable enzyme and metabolic functions^5,6^, biomass accumulation, and DNA replication to ensure cell survival and proliferation^7,8^. Additionally, biosynthesis may place cells under nutrition or redox stresses, and metabolic rewiring must occur to address such challenges to maintain cellular viability and functions^1,8,9^. As such, manipulation of other metabolic pathways or cellular processes might lead to an unexpected improvement in the biosynthesis of the desired product due to distant effects of genetic modulations or unknown regulatory interactions^10–12^. Thus, to construct a superior microbial cell factory for enhanced product biosynthesis, it is highly desirable to perform genome-wide identification of beneficial gene targets in complex intracellular interaction networks that can be reengineered for this purpose.

Free fatty acids (FFAs) are feedstocks for the manufacture of detergents, lubricants, cosmetics, and pharmaceutical ingredients^13^. By engineering metabolic pathways, FFAs production was enhanced in various microorganisms, such as the model microorganisms *Escherichia coli*^14^ and *Saccharomyces cerevisiae*^15^ and the oleaginous microorganisms *Yarrowia lipolytica*^16^ and *Rhodococcus opacus*^17^. Taking *E. coli* as an example, overexpression of key enzymes in the FFA biosynthetic pathway, such as acetyl-coenzyme A (CoA) carboxylase^18,19^ and fatty acyl-ACP thioesterase^18–21^, or deletion of key genes in the beta-oxidation pathway, such as the *fadD*^18–21^ or *fadE*^20,21^ gene, was implemented to improve FFAs production. In particular, optimal combinatorial expression of 15 crucial genes in the FFA biosynthetic pathway with *fadD* deletion in *E. coli* BL21 resulted in a titer of 8.6 g L^−1^ FFAs in fed-batch cultivation with glucose as the carbon source^14^. Despite these efforts, further improvement of FFAs production is hampered by the limited understanding of cellular rewiring mechanism and linkage between FFA biosynthesis and other cellular processes^9,22^. In addition, it remains difficult to systematically identify beneficial gene targets using laborious genome engineering methods, such as homologous recombination-based gene knockout^23^. These hurdles severely restrict the scope and speed of engineering microbial hosts to obtain a superior overproducer.

CRISPR interference (CRISPRi) enables sequence-specific repression of gene transcription by pairing dCas9 with a synthetic sgRNA^24,25^. CRISPRi can be easily designed and readily implemented, enabling rapid screening of beneficial knockdown gene targets. As a promising tool for gene modulation, CRISPRi has been generally used for improved biosynthesis of desired products in *E. coli*^26–29^ and many other microbial hosts, such as *Corynebacterium glutamicum*^30^ and cyanobacteria of the genus *Synechocystis*^31^. However, in most of these cases, the candidate gene targets were restricted to direct competitive pathways^27–31^, which cannot fully unleash the cellular potential for biochemical overproduction^9,22,32^. As an important supplementary method, omics analysis of differentially performing strains allows the acquisition of comprehensive data related to the mechanisms of cellular metabolism^10,32^, providing additional available clues regarding candidate gene targets in complex intracellular interaction networks for enhanced biosynthesis of the desired product^11,33^.

Here, we take advantage of CRISPRi to readily and rapidly downregulate gene expression and omics analyses to identify potential gene targets in cellular interaction networks, enabling rapid, systematic, and effective identification of chromosomal gene targets that can be engineered for enhanced production of FFAs in *E. coli*. Upon CRISPRi-mediated gene repression, we first identify beneficial genes that are directly related to the FFA metabolism. Next, transcriptomic and proteomic analyses are performed for three recombinant FFAs-overproducing strains and a control strain, and beneficial genes in cellular networks are further identified. Upon combinatorial modulation of the identified genes, the engineered strain *ihfA*^L−^-*aidB*^+^-*ryfA*^M−^-*gadA*^H−^ produces 30.0 g L^−1^ FFAs (0.689 g L^−1^ h^−1^ productivity) in fed-batch fermentation. We speculate that the high titer of FFAs obtained in the *ihfA*^L−^-*aidB*^+^-*ryfA*^M−^-*gadA*^H−^ strain benefits from the enhanced cellular stress responses, which protect cells from cytotoxic byproducts during the course of microbial fermentation.

## Results

### Identification of beneficial gene targets in the pathways related to FFA metabolism using the CRISPRi system

**Fig. 1:**
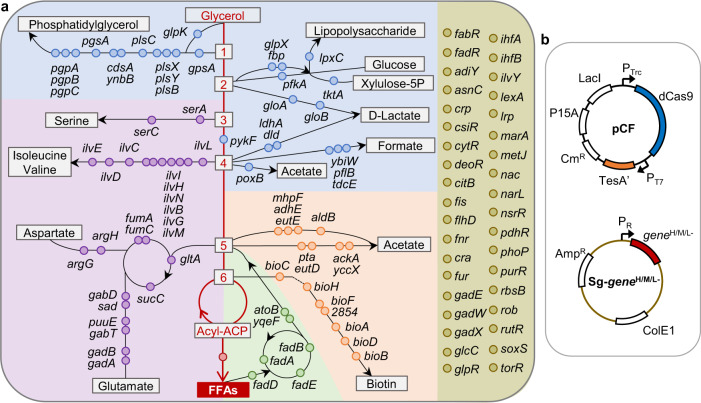
Gene targets related to FFA metabolism that were selected for modulation by the CRISPRi system. **a** Schematic of the metabolic or regulatory pathways related to FFA biosynthesis in *E. coli* BL21(DE3), which were classified into five modules, namely, upstream carbon flux diversion, downstream carbon flux diversion, amino acid metabolism, beta-oxidation, and transcription factor, represented in blue, orange, purple, green, and golden backgrounds, respectively. The 108 candidate genes in the above five modules are marked with blue, orange, purple, green, and golden dots, respectively (see Supplementary Data 1). *2854* represents the gene numbered ECD_02854 in BL21(DE3). The fatty acid biosynthetic pathway is shown as a red line, from glycerol to acyl-ACP and conversion to FFAs by TesA′ (encoded by the *tesA*′gene marked with a red dot). The key metabolites glycerone-P, glyceraldehyde-3P, glycerate-3P, pyruvate, acetyl-CoA, and malonyl-ACP are represented as numbers 1 to 6, respectively. **b** Plasmid constructs for the expression of *tesA*′, *dCas9* and the sgRNAs *gene*^H/M/L−^. Cm^R^, chloramphenicol-resistance gene; Amp^R^, ampicillin-resistance gene; P15A and ColE1, replication origin.

We employed glycerol as the carbon source for the biosynthesis of FFAs. In *E. coli*, glycerol is catalyzed to the key intermediates glyceraldehyde-3P, pyruvate, acetyl-CoA, and malonyl-ACP, in that order, and then converted to acyl-ACP through the fatty acid biosynthetic pathway (Fig. 1a). However, these intermediates are also involved in other metabolic pathways, resulting in the diversion of carbon flux away from FFAs. Expression of the truncated fatty acyl-ACP thioesterase TesA′ (with the leader sequence deleted) could release FFA intermediates from ACP in the cytosol^34^. Nevertheless, synthetic FFAs are degraded naturally by the beta-oxidation process. To channel carbon flux toward the production of FFAs and repress the degradation of FFAs, we chose 108 chromosomal genes in a hypothesis-driven manner for modulation by CRISPRi. The 108 targets comprise 26 genes in the module of upstream carbon flux diversion (those in the competitive pathways consuming glycerone-P, glyceraldehyde-3P, or pyruvate); 15 genes in the module of downstream carbon flux diversion (those in the competitive pathways consuming acetyl-CoA or malonyl-ACP); 24 genes in the module of amino acid metabolism that consume glycerate-3P, pyruvate, or acetyl-CoA; 6 genes in the module of beta-oxidation that degrade FFAs; and 37 genes in the module of transcription factors that regulate multiple pathways or cellular physiology (Fig. 1a and Supplementary Data 1).

**Fig. 2:**
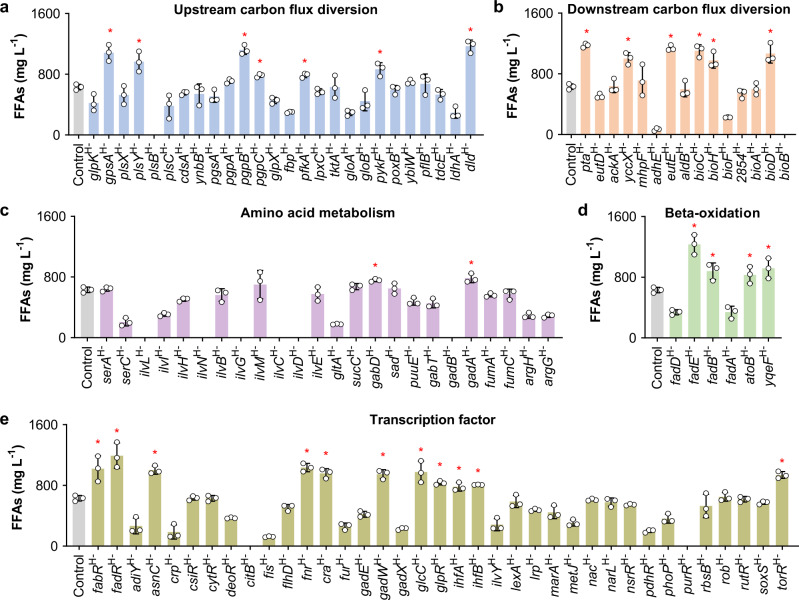
CRISPRi-based identification of beneficial genes for FFAs production. All 108 genes in the upstream carbon flux diversion module (**a**), downstream carbon flux diversion module (**b**), amino acid metabolism module (**c**), beta-oxidation module (**d**), and transcription factor module (**e**) were repressed with high efficiency. Asterisk represents the strain in which the FFAs titer increased by over 20% compared with that in the Control strain (631 mg L^−1^), and the targeted genes are referred to as beneficial genes. The titers were obtained in batch cultivation of 30 g L^−1^ glycerol. Data are presented as mean ± SD (*n* = 3 biological replicates). Source data are provided as a Source Data file.

To facilitate target identification, we constructed a library of 108 synthetic sgRNAs that represses the expression of the 108 chromosomal genes in the above five modules with high efficiency^24^. The P_Trc_ promoter was selected to control the expression of catalytically dead Cas9 (dCas9) in CRISPRi, which could achieve the expected gene repression without affecting cell growth (Supplementary Fig. 1) (see details in the Supplementary Note 1). Plasmid pCF expressing TesA′ and dCas9 was transformed into the BL21(DE3) strain to generate the starting strain CF. We introduced plasmids harboring each of the 108 synthetic sgRNAs individually into the CF strain to implement gene perturbation (Fig. 1b). Plasmid Sg-0 expressing sgRNA0 without the target site complementary sequence was introduced into the CF strain to generate the Control strain, which did not modulate any chromosomal genes and produced 631 mg L^−1^ FFAs. Any CRISPRi-engineered strain that could increase the FFAs titer by over 20% compared with the Control strain was sorted out. As a result, 30 beneficial targets were identified, and the *fadE*^H−^ and *fadR*^H−^ strains achieved FFAs titers of 1232 and 1193 mg L^−1^, which were 95% and 89% higher than that of the Control strain, respectively (Fig. 2).

The identified beneficial genes were mostly distributed in the modules of upstream carbon flux diversion (Fig. 2a), downstream carbon flux diversion (Fig. 2b), beta-oxidation (Fig. 2d), and transcription factors (Fig. 2e). This result suggested that reducing the carbon flux to the byproducts phosphatidylglycerol (*gpsA*, *plsY*, *pgpB*, and *pgpC*), D-lactate (*dld*), acetate (*pta*, *yccX*, and *eutE*), and biotin (*bioC*, *bioH*, and *bioD*), or blocking fatty acid degradation (*fadE*, *fadB*, *atoB*, and *yqeF*) contributed to the enhanced production of FFAs. Reducing the expression of a few transcription factors (namely, *fabR*, *fadR*, *asnC*, *fnr*, *cra*, *gadW*, *glcC*, *glpR*, *ihfA*, *ihfB*, and *torR*) also enhanced the production of FFAs (Fig. 2e), which might benefit from the global regulation of gene expression. However, inhibiting amino acid biosynthetic pathways seemed unfavorable for FFAs production, as most of the engineered strains exhibited reduced titers of FFAs (Fig. 2c), and some of these strains were even unable to grow (Supplementary Fig. 2c). A possible reason is that while these amino acids were necessary for cell viability, the reduced amount of amino acids obtained upon CRISPRi perturbation was insufficient to maintain normal cell physiology.

Among the 30 beneficial gene targets, 20 (*gpsA*, *plsY*, *pgpB*, *pgpC*, *dld*, *yccX*, *bioC*, *bioH*, *bioD*, *gabD*, *gadA*, *asnC*, *fnr*, *cra*, *gadW*, *glcC*, *glpR*, *ihfA*, *ihfB*, *torR*) have not been engineered for FFAs production in previous studies. We found three favorable metabolic pathways that have not been previously modulated for FFAs production, namely, the biotin, phosphatidylglycerol, and glutamate biosynthetic pathways (Figs. 1a, 2). Even in the previously modulated pathways, we identified beneficial gene targets that have not been engineered for FFAs production in previous studies, such as *dld* in the lactate biosynthetic pathway and *yccX* and *eutE* in the acetate formation pathway (Figs. 1a, 2). Additionally, we found that the high repression of *pta* in the acetate formation pathway (strain *pta*^H−^) increased FFAs production by 86% (Fig. 2b), while deletion of this gene in a previous study had a negative effect on FFAs production^35^. Collectively, these results demonstrate that CRISPRi is useful for the systematic identification of beneficial targets that can be engineered for improved biosynthesis of the desired product.

**Fig. 3:**
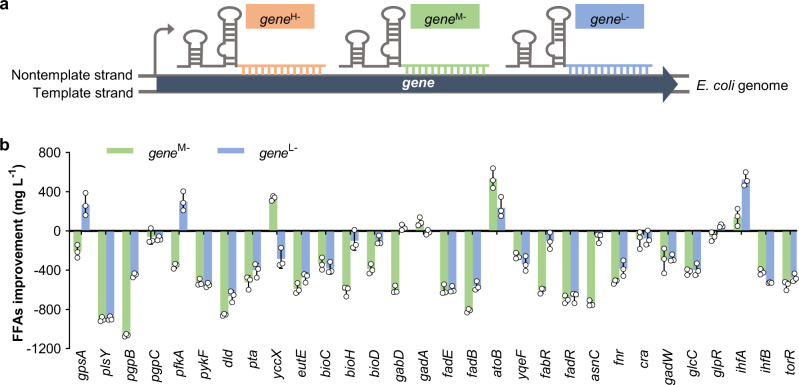
Tuning of the repression of beneficial genes for the improvement of FFAs production. **a** Design of sgRNAs with high (*gene*^H−^), medium (*gene*^M−^), or low (*gene*^L−^) repression efficiency toward the target gene by binding the nontemplate DNA strand at the initial, middle, or terminal region, respectively. **b** Improvement of the FFAs titer upon repressing genes with medium or low efficiency compared with the titer obtained upon repressing genes with high efficiency. The gene targets were the genes marked with an asterisk in Fig. 2. The titers were obtained in batch cultivation of 30 g L^−1^ glycerol. Data are presented as mean ± SD (*n* = 3 biological replicates). Source data underlying (**b**) are provided as a Source Data file.

Fine tuning of gene repression is enormously important for improving the biosynthesis of desired products. Regarding the genes in the competitive pathways, the gene repression level needs to be fine-tuned so that the target genes can still be expressed to a certain degree to maintain cellular viability while resources are maximally redirected toward the target metabolite^26,27,29^. A few approaches have been developed to tune the efficiency of CRISPRi-mediated gene repression^24^. Truncation of the base-pairing region of sgRNA from the 5′ end could severely decrease the repression efficiency; however, it is difficult to achieve a medium repression level^24^. A single mismatch in the seed region or the other 8-nt sequence of the base-pairing region could achieve low or medium repression efficiency, respectively, but the mismatch in the seed region might confer off-target effects^24,36^, thus failing to repress the desired target. Many studies have demonstrated that the repression level in gene expression and the target distance from the transcription start site generally had an inverse relationship^24,37,38^. Most recently, bases surrounding the protospacer adjacent motif (PAM) on the target sequence were also shown to affect the CRISPRi efficiency^39^. We here tuned the repression of each beneficial gene with medium or low efficiency by applying the sgRNA *gene*^M−^ or *gene*^L−^, binding the nontemplate DNA strand at the middle or terminal region, respectively (Fig. 3a). Plasmids harboring each of the synthetic sgRNAs were thus individually introduced into the starting strain CF, and FFAs production of the corresponding recombinant strains was determined. For example, in strain *gpsA*^L−^, the sgRNA *gpsA*^L−^ was expressed, and the *gpsA* gene was repressed with low efficiency. The results showed that the *gpsA*^L−^, *pfkA*^L−^, *yccX*^M−^, *gabD*^L−^, *gadA*^M−^, *atoB*^M−^, *atoB*^L−^, *glpR*^L−^, *ihfA*^M−^, and *ihfA*^L−^ strains produced higher FFAs titers than strains with the corresponding gene repressed with high efficiency (Fig. 3b), and the *gpsA*^L−^, *yccX*^M−^, *atoB*^M−^, and *ihfA*^L−^ strains achieved the highest FFAs titers, which were > 100% higher than that of the Control strain (Supplementary Fig. 3). These results suggested the importance of fine-tuning gene expression in the production of target metabolites.

### Identification of potential targets in the cellular network via proteomic and transcriptomic analyses

**Fig. 4:**
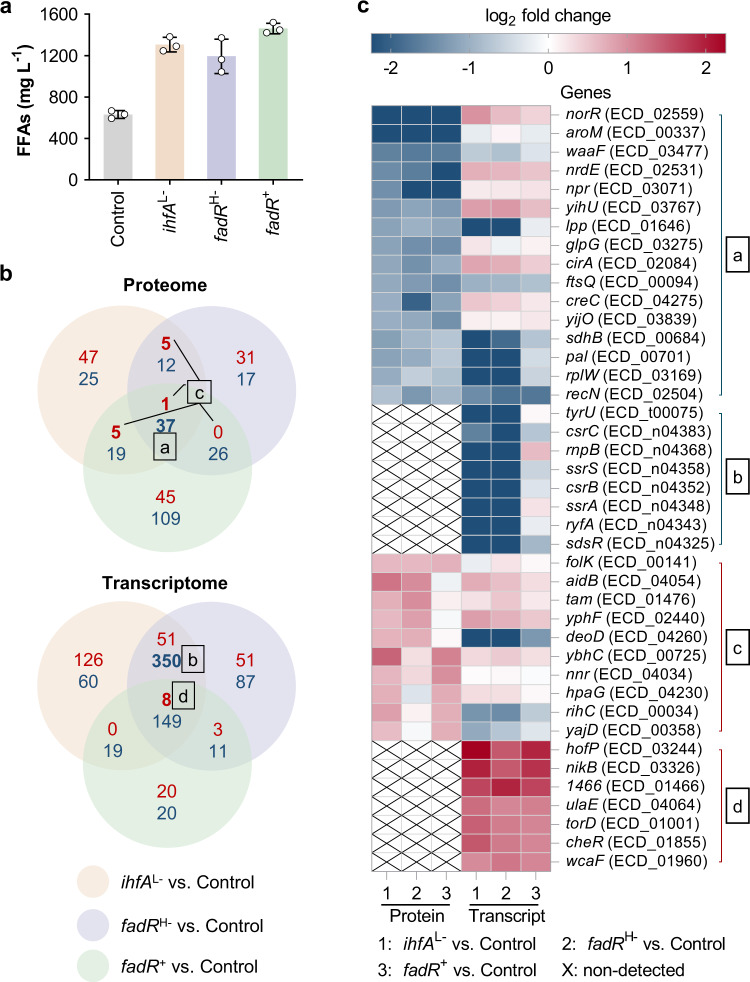
Proteomic and transcriptomic analyses for exploration of available targets for FFAs production. **a** FFAs titer of the overproducing strains *ihfA*^L−^, *fadR*^H−^, and *fadR*^+^ and the Control strain. The titers were obtained in batch cultivation of 30 g L^−1^ glycerol. These strains were applied to the omics analyses. Data are presented as mean ± SD (*n* = 3 biological replicates). **b** Venn diagram of differentially expressed genes. Blue numbers, genes expressed with decreased abundance; red number, genes expressed with increased abundance. Genes in sets a, b, c, and d had decreased abundance at the protein level, decreased abundance at the transcript level, increased abundance at the protein level, and increased abundance at the transcript level, respectively, in at least two FFAs-overproducing strains compared with the Control strain. **c** Abundance of the selected candidate genes at the protein and transcript levels. *1466* represents the gene numbered ECD_01466 in BL21(DE3). Genes in sets a, b, c, and d were selected from sets a, b, c, and d in (**b**). Source data are provided as a Source Data file.

To further explore potential gene targets that could facilitate FFAs production, we focused on the strains with high production of FFAs. In the above studies on CRISPRi-mediated genetic perturbation, the six engineered strains (*fadE*^H−^, *fadR*^H−^, *yccX* ^M−^, *atoB*^M−^, *gpsA*^L−^, and *ihfA*^L−^) achieved the highest FFAs titers (Fig. 2 and Supplementary Fig. 3), which were > 89% higher than that of the Control strain. Among these gene targets, *fadR* encodes the transcription factor FadR, which regulates the processes of fatty acid biosynthesis, degradation, and transport^40^; *ihfA* encodes the alpha subunit of integration host factor, which affects many cell functions (including DNA replication, recombination, and transcription) and influences gene expression on a global scale^41^; and the other four genes are involved in the competitive pathways of FFA biosynthesis (*yccX* and *gpsA*) and the beta-oxidation pathway (*fadE* and *atoB*). Considering the role of transcription factors in the extensive regulation of metabolic processes, we selected the *fadR*^H−^ and *ihfA*^L−^ strains for further analysis. Since overexpression of FadR could significantly enhance FFAs production^40^, we constructed a FadR-overexpressing strain (*fadR*^+^) that could produce a high titer of FFAs (1462 mg L^−1^) (Fig. 4a). Although these three FFAs-overproducing strains have different genotypes, they might have convergent regulatory responses that facilitate FFA biosynthesis. Thus, these three engineered strains (*ihfA*^L−^, *fadR*^H−^ and *fadR*^+^) and the Control strain were applied for proteomic and transcriptomic analyses to investigate the potential target genes and their underlying mechanisms in FFAs overproduction.

Cells were sampled at the early stationary phase (24 h after IPTG induction) (Supplementary Fig. 4). Hundreds of proteins and transcripts were differentially abundant in *ihfA*^L−^ vs. Control, *fadR*^H−^ vs. Control, and *fadR*^+^ vs. Control (Supplementary Figs. 5 and 6). The results indicated that FFAs overproduction might be affected not only by the metabolic processes in which the modulated gene is directly involved, but also by secondary cellular regulatory responses to genetic perturbation. To identify common rewiring in these FFAs-overproducing strains, we plotted the differentially expressed genes of the three strain pairs in a Venn diagram (Fig. 4b). Considering the poor correlation between the proteome and the transcriptome, the candidate gene targets were selected based on their significantly differential abundance at the protein level (a and c) or the transcript level (b and d) (Fig. 4b) (see details in the Supplementary Note 2). As a result, 41 targets were selected, and their differential abundances at the protein and transcript levels are summarized in Fig. 4c. We found that these 41 genes included none of the 108 genes that we first selected from the genes related to FFA metabolism. Although most of the 108 genes were expressed with decreased abundance (Supplementary Data 2), none met the selection standards for significantly differential abundance (fold change < 0.67 at the protein level) in three FFAs-overproducing strains. Thus, these 41 gene targets were newly identified and were not directly related to FFA biosynthesis.

**Fig. 5:**
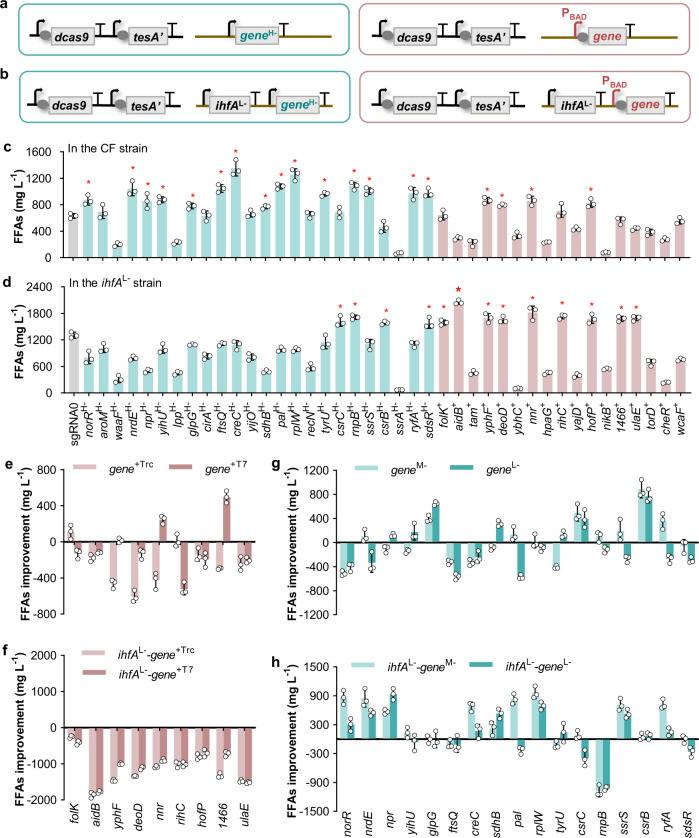
Reverse engineering of the candidate targets derived from omics analyses for identification of beneficial genes and tuning of gene expression for enhanced FFAs production. Schematic of cassettes used to repress or overexpress the target genes in the CF strain (**a**), or in the *ihfA*^L−^ strain (**b**). High repression was achieved by applying sgRNA *gene*^H−^, and overexpression was enabled by the P_BAD_ promoter. TesA′, dCas9, and sgRNAs were controlled by the P_T7_, P_Trc_, and P_R_ promoters, respectively. Effect of perturbation of the target genes on FFAs production in the CF strain (**c**) or in the *ihfA*^L−^ strain (**d**). Asterisk represents the strain in which the FFAs titer increased by over 20% compared with that in the reference strain in each bar chart, and the targeted genes are referred to as beneficial genes. Effect of tuning the expression of beneficial genes on FFAs production in the CF strain (**e**, **g**) or in the *ihfA*^L−^ strain (**f**, **h**). The overexpression was tuned by utilizing the P_Trc_ or P_T7_ promoter, and the repression was tuned by applying the sgRNA *gene*^M−^ or *gene*^L−^. Red bar, overexpression; green bar, repression. The titers were obtained in batch cultivation of 30 g L^−1^ glycerol. Data are presented as mean ± SD (*n* = 3 biological replicates). Source data underlying (**c**–**h**) are provided as a Source Data file.

To test the function of these 41 candidate targets in FFAs production, the corresponding modulation of each gene was conducted based on the differential abundance at the protein level (the a and c sets in Fig. 4c) or the transcript level (the b and d sets in Fig. 4c). We first applied CRISPRi to repress gene expression with high efficiency or utilized the P_BAD_ promoter to upregulate gene expression (Fig. 5a, c). Considering that the selected genes were all differentially expressed in the *ihfA*^L−^ vs. Control strain pair (Fig. 4c), these targets were also manipulated in the *ihfA*^L−^ strain to test their effects on FFAs production (Fig. 5b, d). The plasmids carrying the cassettes for each candidate gene were transformed into the starting strain CF or the *ihfA*^L−^ strain (Fig. 5a, b). An FFA assay of the resulting strains showed that modulation of 26 of the identified genes enhanced FFAs production by over 20% in comparison to that in the Control or *ihfA*^L−^ strain (Fig. 5c, d). In particular, the highest FFAs production was obtained in the *ihfA*^L−^-*aidB*^+^ strain, in which the *ihfA* gene was repressed with low efficiency and the *aidB* gene was overexpressed under the P_BAD_ promoter. The titer reached up to 2052 mg L^−1^, which was 57% and 225% higher than the titers in the *ihfA*^L−^ and Control strains, respectively (Fig. 5d). In summary, 26 (*norR*, *nrdE*, *npr*, *yihU*, *glpG*, *ftsQ*, *creC*, *sdhB*, *pal*, *rplW*, *tyrU*, *crsC*, *rnpB*, *ssrS*, *csrB*, *ryfA*, *sdsR*, *folk*, *aidB*, *yphF*, *deoD*, *nnr*, *rihC*, *hofP*, ECD_01466, and *ulaE*) newly identified genes were determined to be beneficial targets that enhanced FFAs production.

Considering the significance of the gene expression level for improving product biosynthesis, we further tuned the expression level of each beneficial gene. We replaced the P_BAD_ promoter with the stronger promoter P_trc_ or P_T7_ (P_T7 > _P_trc > _P_BAD_)^42^ to control the overexpression of the beneficial genes. The results showed that only three strains exhibited increased FFAs production compared with strains with the corresponding gene controlled by the P_BAD_ promoter (Fig. 5e, f and Supplementary Fig. 7a, b). Additionally, we utilized sgRNAs that bind the nontemplate strand at the middle or terminal region to repress the beneficial gene with medium or low efficiency (Fig. 3a). We found that more than half of the engineered strains with the tuned gene repression level obtained further improved FFAs production compared with the strains with the corresponding gene repressed with high efficiency (Fig. 5g, h and Supplementary Fig. 7c, d).

**Fig. 6:**
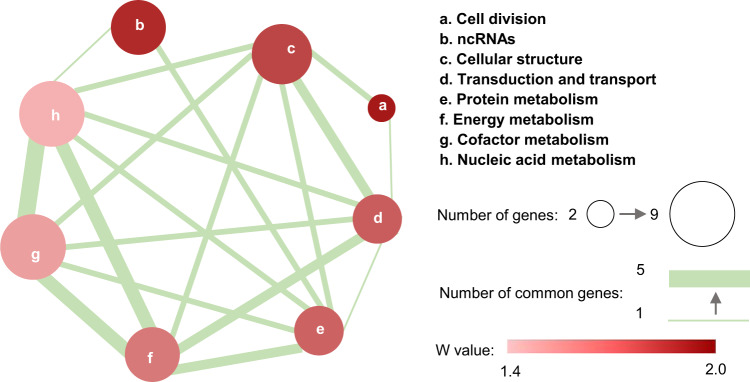
Functional network plot of the beneficial genes derived from omics analyses. The size of the node represents the number of genes within the function set. The node color represents the W value of the function set. The thickness of the line indicates the number of genes overlapping between two function sets. The W value was calculated by averaging the maximum FFAs ratio of the engineered strains corresponding to each gene in the function set. Genes and the FFAs ratio are shown in Supplementary Data 3. Source data are provided as a Source Data file.

From the candidate gene targets provided by omics analyses, 26 beneficial genes that could improve FFAs production were verified (Fig. 5). However, these newly identified beneficial gene targets were not present in the metabolic pathways directly related to FFA metabolism. To mine the related regulatory mechanisms for FFAs production, the functions of the 26 beneficial genes were analyzed by using the NCBI, KEGG, UniProt, and Gene Ontology database, and summarized into eight sets (Fig. 6 and Supplementary Data 3). Based on the FFAs titer of the corresponding engineered strains, the weight value (W value) of each set was calculated to characterize the effects on the enhancement of FFAs production. The eight sets were arranged in descending order of W value as follows: cell division; ncRNAs; cellular structure; transduction and transport; protein metabolism; energy metabolism; cofactor metabolism; and nucleic acid metabolism (Fig. 6 and Supplementary Data 3). Surprisingly, processes such as cell division, signal transduction, protein metabolism, and nucleic acid metabolism are seemingly irrelevant to FFA metabolism and thus generally inaccessible to target in the hypothesis-driven methods.

Then, we further dissected the underlying connections between these enriched cellular processes and FFAs production. High repression of *ftsQ* or *pal* increased the intracellular FFAs concentration by 88% or 113%, respectively, compared with that in the Control strain (Supplementary Fig. 8), which might be associated with the increased cell volume for storing FFAs, since inactivation of either of the two genes could result in a deficiency in cell division and lead to the formation of long multiseptated cell chains^43,44^. Beneficial effects of the downregulation of membrane proteins (such as those encoded by the genes *glpG*, *ftsQ*, *creC*, and *pal*) might be associated with microbial tolerance to FFAs^45^. Downregulation of *creC*, a sensor for the early response to phosphate starvation^46^, was favorable for enhancing FFAs production, which is consistent with the promotive effects of cultivation under phosphate limitation^47^. The effect of *npr* downregulation and *yphF* upregulation may be associated with the reduction in nitrogen assimilation^48^ and the elevation of carbohydrate uptake^49^, indicating a regulatory role of nitrogen and carbon utilization in enhancing FFAs production. Repression of *rplW* (encoding 50 S ribosomal subunit protein L23), *tyrU* (encoding tRNA-Tyr), and *rnpB* (encoding M1 RNA, the catalytic component of the tRNA-processing enzyme RNase P) was associated with the inhibition of protein synthesis, which increased the partitioning of total carbon from protein to lipids^11^. Although seemingly irrelevant to FFA metabolism, modulating these genes could markedly boost FFAs production due to the complex interactions of cellular networks. These findings provide insights into linkages between cell functions and product biosynthesis, providing avenues for the construction of a superior microbial cell factory for biochemical overproduction.

### Enhancing FFAs production by combinatorial genetic perturbation

**Fig. 7:**
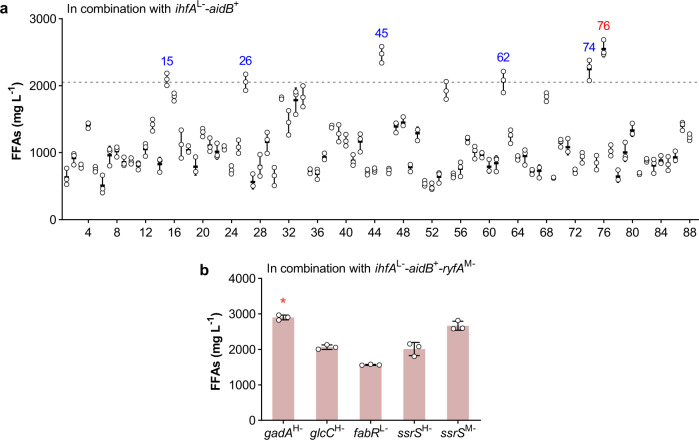
Combinatorial genetic perturbation for improved FFAs production. **a** FFAs production of *ihfA*^L−^-*aidB*^+^ in combination with each beneficial sgRNA. The gray line represents the FFAs titer of the *ihfA*^L−^-*aidB*^+^ strain. Numbers 1 to 88 represent the sgRNA *gpsA*^H−^, *plsY*^H−^, *pgpB*^H−^, *pgpC*^H−^, *pfkA*^H−^, *pykF*^H−^, *dld*^H−^, *pta*^H−^, *yccX*^H−^, *eutE*^H−^, *bioC*^H−^, *bioH*^H−^, *bioD*^H−^, *gabD*^H−^, *gadA*^H−^, *fadE*^H−^, *fadB*^H−^, *atoB*^H−^, *yqeF*^H−^, *fabR*^H−^, *fadR*^H−^, *asnC*^H−^, *fnr*^H−^, *cra*^H−^, *gadW*^H−^, *glcC*^H−^, *glpR*^H−^, *ihfB*^H−^, *torR*^H−^, *gpsA*^M−^, *yccX*^M−^, *bioC*^M−^, *gadA*^M−^, *atoB*^M−^, *cra*^M−^, *glpR*^M−^, *gpsA*^L−^, *pfkA*^L−^, *pta*^L−^, *bioH*^L−^, *bioD*^L−^, *gabD*^L−^, *gadA*^L−^, *atoB*^L−^, *fabR*^L−^, *asnC*^L−^, *cra*^L−^, *glp*R^L−^, *norR*^H−^, *nrdE*^H−^, *npr*^H−^, *yihU*^H−^, *glpG*^H−^, *ftsQ*^H−^, *creC*^H−^, *sdhB*^H−^, *pal*^H−^, *rplW*^H−^, *tyrU*^H−^, *csrC*^H−^, *rnpB*^H−^, *ssrS*^H−^, *csrB*^H−^, *ryfA*^H−^, *sdsR*^H−^, *norR*^M−^, *nrdE*^M−^, *glpG*^M−^, *creC*^M−^, *pal*^M−^, *rplW*^M−^, *csrC*^M−^, *rnpB*^M−^, *ssrS*^M−^, *csrB*^M−^, *ryfA*^M−^, *sdsR*^M−^, *npr*^L−^, *yihU*^L−^, *glpG*^L−^, *creC*^L−^, *sdhB*^L−^, *rplW*^L−^, *tyrU*^L−^, *csrC*^L−^, *rnpB*^L−^, *ssrS*^L−^, and *csrB*^L−^, respectively. Blue number, FFAs titer in the corresponding strain was increased compared with that in the *ihfA*^L−^-*aidB*^+^ strain; red number, the corresponding strain obtained the highest FFAs titer, which was applied for the next round of combination. **b** FFAs production of *ihfA*^L−^-*aidB*^+^-*ryfA*^M−^ in combination with *gadA*^H−^, *glcC*^H−^, *fabR*^L−^, *ssrS*^H−^, or *ssrS*^M−^. Asterisk represents the strain that produced the highest FFAs titer, and this strain was applied to the next fed-batch fermentation. The titers were obtained in batch cultivation of 30 g L^−1^ glycerol. Data are presented as mean ± SD (*n* = 3 biological replicates). Source data are provided as a Source Data file.

In the above studies on the modulation of candidate targets, we identified 56 beneficial genes (Figs. 2, 5c, d) and verified the corresponding beneficial sgRNAs that could be expressed to improve FFAs production (Fig. 2, Supplementary Fig. 3, Fig. 5c, d, and Supplementary Fig. 7c, d). All these beneficial sgRNAs are listed in Supplementary Data 4. The resulting strain *ihfA*^L−^-*aidB*^+^ achieved the highest FFAs titer of 2,052 mg L^−1^ (Fig. 5d). To further enhance FFAs production, we performed combinatorial multiplex perturbation of these beneficial genes. First, FFAs production of the *ihfA*^L−^-*aidB*^+^ strain in combination with each of the 88 beneficial sgRNAs was investigated (Fig. 7a). The sgRNAs *ihfA*^H−^, *ihfA*^M−^, and *ihfA*^L−^ were excluded from the combination, as the target gene *ihfA* was modulated in the *ihfA*^L−^-*aidB*^+^ strain. The results showed that *ihfA*^L−^-*aidB*^+^ in combination with *gadA*^H−^ (No. 15), *glcC*^H−^ (No. 26), *fabR*^L−^ (No. 45), *ssrS*^H−^ (No. 62), *ssrS*^M−^ (No. 74), or *ryfA*^M−^ (No. 76) improved FFAs production compared with the *ihfA*^L−^-*aidB*^+^ strain. Among these combinations, *ihfA*^L−^-*aidB*^+^ in combination with *ryfA*^M−^ (No. 76) obtained the highest FFAs titer of 2,550 mg L^−1^. Then, the corresponding strain *ihfA*^L−^-*aidB*^+^-*ryfA*^M−^ was utilized for further combination with the other five beneficial sgRNAs (*gadA*^H−^, *glcC*^H−^, *fabR*^L−^, *ssrS*^H−^, and *ssrS*^M−^) (Fig. 7b). The final resulting strain *ihfA*^L−^-*aidB*^+^-*ryfA*^M−^-*gadA*^H−^ (*ihfA*^L−^-*aidB*^+^-*ryfA*^M−^ in combination with *gadA*^H−^, overexpressing *aidB* via the P_BAD_ promoter and repressing *ihfA*, *ryfA*, and *gadA* with low, medium, and high efficiency, respectively) produced 2,901 mg L^−1^ FFAs (Fig. 7b), the highest titer among all our engineered strains, 41% and 360% higher than that of the *ihfA*^L−^-*aidB*^+^ and Control strains, respectively. These results strongly demonstrate the significance of these identified beneficial targets for FFAs overproduction.

To elucidate the mechanism of FFAs overproduction in the *ihfA*^L−^-*aidB*^+^-*ryfA*^M−^-*gadA*^H−^ strain, we analyzed the effect of modulating the target genes. *ihfA* encodes the alpha subunit of the integration host factor, which affects DNA replication, recombination, and transcription^41^. Low repression of *ihfA* induces comprehensive regulation of gene expression (including *aidB* and *ryfA*) that promotes FFAs production (Supplementary Figs. 5, 6 and Fig. 5c, d). *aidB* encodes a DNA repairing protein involved in adaptive response of cells. Overexpression of *aidB* protects cells from cytotoxic effects of acidified cytoplasm or alkylating agents (released as byproducts of cellular metabolism)^50,51^. *ryfA* encodes a small RNA (sRNA) with unknown functions. Nevertheless, bacterial sRNAs have been characterized as native mediators of stress responses^52–54^. Repression of *ryfA* decreases membrane damage under stress conditions^55^. *gadA* encodes glutamate decarboxylase A, which catalyzes the decarboxylation of glutamate and produces carbon dioxide. sgRNA targeting *gadA* would also inhibit the expression of the downstream gene *gadX* (encoding an activator of the glutamate decarboxylase system) in the same operon^56^, resulting in coordinated repression of *gadA*. Repression of *gadA* could decrease the production of carbon dioxide, a toxic byproduct inhibitory to cellular metabolism^57^. Overall, we speculate that genes modulation in the *ihfA*^L−^-*aidB*^+^-*ryfA*^M−^-*gadA*^H−^ strain improves the cellular stress responses to protect cells from toxic effects and support the cellular phenotype of FFAs overproduction. In pursuit of a high titer of the desired product, microbial cells often encounter harsh and complex stressors, such as toxic byproducts and polytropic environments^1^. Thus, enhancing stress responses to reinforce microbial stress tolerance is a promising method to improve biochemicals production.

### FFAs production by fed-batch fermentation

**Fig. 8:**
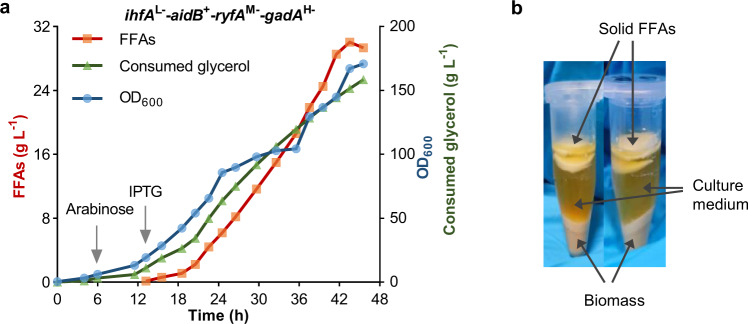
Fed-batch fermentation of the *ihfA*^L−^-*aidB*^+^-*ryfA*^M−^-*gadA*^H−^ strain for FFAs production in a 5 L bioreactor. **a** Time courses of cell growth, glycerol consumption, and FFAs production of the *ihfA*^L−^-*aidB*^+^-*ryfA*^M−^-*gadA*^H−^ strain during fed-batch fermentation (Batch 1). The result of a duplicate fermentation (Batch 2) is shown in Supplementary Fig. 9b, exhibiting a similar titer and productivity. **b** Solid FFAs layer after centrifugation. After fermentation, 1 mL of sample was picked up and centrifuged at 5000 × *g* for 15 min. Source data underlying (**a**) are provided as a Source Data file.

To further evaluate the potential of our optimized strain *ihfA*^L−^-*aidB*^+^-*ryfA*^M−^-*gadA*^H−^ in a scaled-up process, fed-batch fermentations were performed in 5 L fermenters using the dO_2_-stat feeding strategy (details described in the “Methods”). The *ihfA*^L−^-*aidB*^+^-*ryfA*^M−^-*gadA*^H−^ strain produced an exceptionally high titer of FFAs (30.0 g L^−1^ titer and 0.689 g L^−1^ h^−1^ productivity) (Fig. 8a) in comparison to both the Control strain (8.6 g L^−1^ titer and 0.231 g L^−1^ h^−1^ productivity) (Supplementary Fig. 9a) and previously published values of FFAs fermentation performance in *E. coli*^58^. Duplicate fermentation of the *ihfA*^L−^-*aidB*^+^-*ryfA*^M−^-*gadA*^H−^ strain resulted in a similar titer and productivity (Supplementary Fig. 9b). Extracellularly secreted FFAs were clearly visible at the top of the culture medium after centrifugation (Fig. 8b). Moreover, floating dead cells or fatty acid particles^58,59^ were precipitated and stuck on the inner wall of the fermenter and on the sensors over the course of the fermentation process (Supplementary Fig. 9c, d), exhibiting an additional amount of FFAs that is difficult to accurately quantify and not included in the FFAs titers.

## Discussion

Genome-scale identification of beneficial gene targets is especially necessary for improving the production of desired products since microbial biosynthesis is subject to sophisticated interactions. In our work, we took advantage of the CRISPRi system and omics analyses to identify beneficial genes for FFAs overproduction from candidate targets that are directly related or seemingly irrelevant to FFA metabolism (Supplementary Fig. 10 and Supplementary Data 1). First, a set of 108 candidate targets was selected in a hypothesis-driven manner based on the competitive or regulatory roles of the genes in FFA biosynthesis (Fig. 1). We identified 30 beneficial genes from these targets via CRISPRi-mediated repression with high efficiency (Fig. 2). We further evaluated each of these 30 genes at two repression levels and obtained strains with further enhanced FFAs production (Fig. 3b). Considering the high titer of FFAs and the extensive regulation of genetic networks, three FFAs-overproducing strains (*ihfA*^L−^, *fadR*^H−^, and *fadR*^+^) that modulated transcription factors and the Control strain were applied for comparative transcriptomic and proteomic analyses, leading to the discovery of a set of 41 candidate targets that were significantly differentially expressed (Fig. 4). From these gene targets, we identified 26 beneficial genes (Fig. 5c, d), which were associated with cell division, ncRNAs, signal transduction, transport, protein metabolism, and nucleic acid metabolism, etc. (Fig. 6). We further evaluated each of these 26 genes at two expression levels and obtained strains with further enhanced FFAs production (Fig. 5e–h). Overall, we identified 56 beneficial genes that could significantly improve FFAs production, 30 of which were directly related to FFA metabolism and 26 of which were seemingly irrelevant to FFAs production. By combinatorial perturbation of the identified genes (repressing *ihfA*, *ryfA*, and *gadA* and overexpressing *aidB*), we obtained the optimized strain *ihfA*^L−^-*aidB*^+^-*ryfA*^M−^-*gadA*^H−^ (Fig. 7), producing 30.0 g L^−1^ FFAs (0.689 g L^−1^ h^−1^ productivity) in fed-batch fermentation (Fig. 8). These titer and productivity of the microbial FFAs production are high in comparison to previous studies (Supplementary Table 1). Our findings shed light on the connection between target metabolite biosynthesis and complex interaction networks, aiding in exploiting the full cellular potential for further improvement in the production of target chemicals. This combined method for identifying beneficial targets and unleashing the cellular potential may be extended to other microbes to optimize the production of other desired chemicals.

In this work, we utilized CRISPRi to readily downregulate gene expression by blocking transcription elongation^24,25^. Several other strategies could also be utilized to achieve gene repression^60,61^. RNA interference (RNAi) can downregulate gene expression by translational inhibition or transcript degradation; however, RNAi is limited to eukaryotes that have the proper host machinery^60^. In bacteria, gene repression can be achieved by knocking down the translation of selected target mRNAs by using synthetic sRNAs^61^. Both CRISPRi and sRNAs can be easily designed and readily implemented with varied efficiency and multiplex combination, enabling rapid screening of beneficial knockdown targets, including genes involved in essential metabolism, for enhanced production of biochemicals. An advantage of CRISPRi is that diverse regions, such as trans-acting small ncRNAs and cis-encoded untranslated regions, can be targeted, enabling the acquisition of additional regulatory knowledge. As a result, several beneficial ncRNAs, such as those encoded by *ryfA* and *ssrS*, were identified in our work, and the corresponding regulation greatly improved FFAs production (Fig. 5).

In the process of target identification, we observed an intriguing phenomenon of FFAs overproduction upon modulation of the *fadR* gene. Overexpression of *fadR* could increase the output of FFAs, as shown in a previous study^40^, which was also verified in our *fadR*^+^ strain in this study (Fig. 4a). Conversely, we found that high repression of *fadR* (*fadR*^H−^) could also enhance FFAs production (Fig. 4a). To reveal the mechanisms underlying FFAs overproduction in the *fadR*^+^ and *fadR*^H−^ strains, we analyzed the data acquired from proteomic and transcriptomic analyses of these two strains compared with the Control strain (Supplementary Fig. 11a, b). FadR is a transcription factor that controls the expression of genes involved in FFA metabolism^40^. We found that the abundance of AccD, FabB, and FabI in the FFA biosynthetic pathway was significantly increased (fold change of 1.96, 2.14, and 1.63, respectively) in *fadR*^+^ vs. Control (Supplementary Fig. 11a). Thus, overexpression of *fadR* strengthened the FFA biosynthetic pathway, resulting in FFAs overproduction in the *fadR*^+^ strain (Supplementary Fig. 11c). Regarding the *fadR*^H−^ strain, we speculated that high repression of *fadR* could enhance cellular responses to reconcile intracellular and environmental stresses, maintaining a functional cellular state to facilitate FFAs overproduction (Supplementary Fig. 11c). On the one hand, we observed that the abundance of several sRNAs, such as those encoded by *ssrS* and *ryfA*, was significantly decreased (fold change < 0.5) in *fadR*^H−^ vs. Control (Supplementary Fig. 11b). Modulation of these sRNAs, native mediators of stress responses, enables the cell to respond to stresses to support the overproduction phenotype^52–54^. On the other hand, we also found that the protein and transcript abundances of *aidB* were increased (fold change of 2.02 and 1.47, respectively) in *fadR*^H−^ vs. Control (Supplementary Fig. 11b). Increased expression of AidB serves to protect cells from the cytotoxic effects of an acidified cytoplasm or alkylating agents^50,51^. In fact, a similar phenomenon wherein both up- and downregulating the expression of a gene could facilitate product biosynthesis has been previously observed, for example, the improved production of lycopene upon overexpressing or deleting the *POX2* gene (encoding acyl-CoA oxidase)^62,63^.

Our study demonstrates that the identification of crucial gene targets is of enormous importance in engineering microbes for enhanced production of desired biochemicals. Further effort should be made for multilevel and large-scale identification of beneficial gene targets in the chromosomes of microbes. For example, the CRISPRa (CRISPR activation) method could be utilized to identify genes that would enhance target chemical production by upregulation of specific genes. CRISPRa enables the activation of endogenous targets by expressing the activator and the dCas9-sgRNA complex^37,64–68^. In bacteria, however, CRISPRa currently has stringent requirements for effective target sites and lacks general functional activators^37,65,66^, which constrain its wide applications. Additionally, interconnected with high-throughput screening of a desired phenotype, it would be better to apply a genome-scale sgRNA library for identifying beneficial targets from the complete list of directly related and seemingly irrelevant genes^69,70^. Future advances in genome-scale technologies and high-throughput screening methods will enable systematic and comprehensive identification of potential chromosomal gene targets, unleashing the full cellular potential for microbial biosynthesis.

## Methods

### Experimental materials

All strains and plasmids used in this study are listed in Supplementary Data 5 and Supplementary Data 6, respectively. All genes targeted in this study are listed in Supplementary Data 1. In consideration of the polar effect of CRISPRi^56^, the operon context of each gene is also summarized in Supplementary Data 1. *E. coli Trans*1-T1 was used for cloning. *E. coli* BL21(DE3) and its derived strains were used for fermentation. Plasmids pACYCDuet-1, YX210, YX212, and YX213 were used as the vectors for the expression of dCas9, GFP, or TesA′. Plasmid Sg-S (with two BsaI restriction sites) was constructed and used as a platform vector for the expression of various sgRNAs. Plasmids Sg-BAD, Sg-Trc, and Sg-T7 were used as the vectors for the expression of chromosome genes. Plasmids, excluding that expressing sgRNAs, were constructed by standard enzyme digestion and ligation. Target site complementary sequences of sgRNAs were designed according to a previous study^25^. Custom-designed spacers were inserted into plasmid Sg-S by one-step Golden Gate assembly^37,71^, allowing rapid construction of plasmids for the expression of sgRNAs targeting any genomic locus of interest. Briefly, the designed forward and reverse primers were annealed to obtain a double-stranded inserted fragment, which could be cleaved by BsaI and ligated into plasmid Sg-S by T4 DNA ligase, resulting in the desired sgRNA expression plasmid Sg-*gene*^H/M/L−^. Primers annealed to custom-designed spacers and primers used to amplify genes from *E. coli* genomic DNA are listed in Supplementary Data 7 and Supplementary Data 8, respectively. DNA sequences used in this study are listed in Supplementary Data 9. The construction of plasmids is presented in Supplementary Fig. 12.

### Culturing conditions and media

To assess FFAs production by different recombinant *E. coli* strains, fermentation was performed in 34 mL glass tubes (180 mm in length and 18 mm in caliber) containing 5 mL of modified M9 medium with oblique and orbital shaking at 220 rpm and 30 °C. Each strain was inoculated from a freshly transformed single colony in LB agar plate to 2 mL of LB medium as seed culture. When cell accumulation reached stationary phase, 1% (V/V) of seed culture was re-inoculated to 5 mL of modified M9 medium in a glass tube. The tube cultures were induced with 1 mM IPTG at an OD_600_ of about 1.0 and allowed to grow for an additional 40 h. When the P_BAD_ promoter was applied, 10 mM arabinose was utilized to induce gene expression at an OD_600_ of 0.5–0.6. This sequential addition of arabinose and IPTG was to avoid the inhibition of the arabinose-inducible promoter P_BAD_ expression system by IPTG^72^, which was also applied in many previous studies^19,73^. Tube cultivations were performed in three biological replicates. For proteomic and transcriptomic analyses, fermentation was implemented in 250 mL unbaffled Erlenmeyer flasks containing 50 mL of modified M9 medium with the identical culturing procedures of tube fermentation. Flask cultivations were performed in two biological replicates. Glycerol, a byproduct of soap manufacturing, is utilized here as a common feedstock for FFAs production in *E. coli* (Supplementary Table 2). Modified M9 medium used for tube and flask fermentation^74^ was as described: 17.1 g L^−1^ Na_2_HPO_4_·12H_2_O, 3 g L^−1^ KH_2_PO_4_, 0.5 g L^−1^ NaCl, 2 g L^−1^ NH_4_Cl, 2 g L^−1^ yeast extract, 30 g L^−1^ glycerol, 0.25 g L^−1^ MgSO_4_·7H_2_O, 11.1 mg L^−1^ CaCl_2_, 10 mg L^−1^ thiamine, and 0.1% (v/v) Triton-X100. 1 mL L^−1^ metal trace stock solution was also supplemented, which contained 27 g L^−1^ FeCl_3_·6H_2_O, 2 g L^−1^ ZnCl_2_, 2 g L^−1^ Na_2_MoO_4_·2H_2_O, 1.9 g L^−1^ CuSO_4_·5H_2_O, and 0.5 g L^−1^ H_3_BO_3_. pH was adjusted to about 7.2 by Tris. If necessary, 34 μg mL^−1^ chloramphenicol or 100 μg mL^−1^ carbenicillin was supplemented.

For fed-batch fermentation, an overnight LB culture (1 mL) of the freshly transformed single colony in 34 mL glass tubes was re-inoculated into 100 mL of modified mineral medium (6 g L^−1^ NH_4_Cl, 8.5 g L^−1^ KH_2_PO_4_, 0.5 g L^−1^ citrate, 5 g L^−1^ yeast extract, 15 g L^−1^ glycerol, 1 g L^−1^ MgSO_4_·7H_2_O, 0.07 g L^−1^ CaCl_2_·2H_2_O, 4 mL L^−1^ metal trace stock solution, and 100 mg L^−1^ thiamine)^74^ with 34 μg mL^−1^ chloramphenicol and 100 μg mL^−1^ carbenicillin in 500 mL unbaffled flasks. When OD_600_ reached 3-4, 200 mL of culture was re-inoculated into a 5 L bioreactor (T&J, China) with 1.8 L of modified mineral medium, 34 μg mL^−1^ chloramphenicol, and 100 μg mL^−1^ carbenicillin. Fermentation temperature was set at 30 °C and pH was controlled at 7 by feeding 6 N ammonium hydroxide via an auto pump. Air flow rate was maintained at around 2 L min^−1^. The dissolved oxygen (dO_2_) concentration was controlled above 30% by agitation cascade (300–800 rpm). When cell density (OD_600_) reached about 6 and 17, 10 mM arabinose (when the P_BAD_ promoter was applied) and 1 mM IPTG were added into the fermentation cell culture, respectively. Feeding medium (2.47 g L^−1^ MgSO_4_·7H_2_O, 500 g L^−1^ glycerol, and 100 g L^−1^ yeast extract) was fed to the fermentation culture when the initial glycerol was almost depleted and the dissolved oxygen showed a sharp increase. The carbon source restriction strategy was carried out by dO_2_ concentration, given that the dO_2_ level immediately spiked once the carbon source was run out. Once the dO_2 _> 60%, the feeding would automatically start at 5 mL min^−1^ until dO_2_ < 60%. Antifoam (Sigma) was added automatically as needed. Broth samples (about 5 mL) were collected at a series of time points to measure cell density and stored at −20 °C for further measurements of glycerol and FFAs.

### GFP fluorescence assay

The fluorescence signal of GFP was used to characterize the repression efficiency of the CRISPRi system. Culturing procedures and induction process of the GFP reporting strains were the same as tube fermentation of the FFAs-producing strains. For each tube, 200 μL of the sample was diluted into the linear range of the detector with phosphate-buffered saline (PBS) at 20 h after IPTG induction. Fluorescence intensity (excitation at 485 nm and emission at 520 nm) and cell density (OD_600_) were detected using 96-well polystyrene plates (black plate with a clear bottom) (Corning Incorporated 3603, USA) and a microplate reader (SpectraMax M2, Molecular Devices, USA). The relative fluorescence intensity was first normalized using OD_600_ and then subtracted that of blank *E. coli* BL21(DE3). Experiments were performed in three biological replicates.

### Metabolite extraction and analysis

FFAs titers in whole-cell culture (only FFAs were measured in this study) were quantified following previously published methods^75^. Specifically, 0.5 mL of cell culture (or an appropriate volume of cell culture diluted to 0.5 mL) was acidified with 50 μL of concentrated HCl, spiked with 120 μg of heptadecanoic acid as internal standard. The cell culture was extracted twice with 0.5 mL of ethyl acetate. The extracted FFAs were then determined using a Thermo Scientific TRACE 1300 gas chromatograph (GC) equipped with a TG-WaxMS A column (30 m × 0.32 mm × 0.25 µm; Thermo Scientific) and a Flame Ionization Detector (FID) operating under constant flow rate of the carrier gas (nitrogen) at 1 mL min^−1^. The following temperature program was used: hold at 50 °C for 1 min, then heat to 245 °C at 30 °C min^−1^ and hold at this temperature for 22.5 min. Individual fatty acid species were qualified by authentic homologous standards and quantified by comparing the peak areas with that of the internal standard using the Chromeleon 7.1 software. Total concentrations of FFAs were calculated as the sum of C_12_ to C_18_ (saturated and monounsaturated).

Glycerol concentration was determined by high-performance liquid chromatography (HPLC) following Waters standard protocols. Briefly, filtered culture supernatants were analyzed by a Waters HPLC system including a Waters e2695 separation module, a Waters 2414 refractive index detector (RID) and an Aminex HPX-87H column (Bio-Rad). The separation was performed through elution with 5 mM H_2_SO_4_ at a flow rate of 0.6 mL min^−1^ at 65 °C for 30 min.

### Proteomic analysis

Cells were harvested by centrifugation at 3000 ×g for 5 min at 24 h after IPTG induction and flash frozen in liquid nitrogen. For protein preparation, cells were resuspended in 600 μL of lysis buffer (0.05 M Tris, pH 8, 1% SDS, and 8 M Urea) and disrupted by ultrasonication for 5 min (cycles with 2 s work and 3 s pause). After centrifugation at 18,000 × *g* and 4 °C for 20 min, the supernatant was reduced with 10 mM dithiothreitol for 1 h at 56 °C, and subsequently alkylated with sufficient iodoacetamide for 1 h at room temperature in the dark. Samples were subjected to protein precipitation by being vortexed with pre-cooled acetone. After centrifugation, the precipitate was collected, dried, and re-dissolved in 600 μL of Urea buffer (0.05 M Tris, pH 8, and 8 M Urea). The protein samples were quantified by the Bradford method. A total of 100 μg of each sample was trypsin-digested overnight. After desalination, the tryptic peptides were lyophilized, re-dissolved in 0.5 M triethylammonium bicarbonate (TEAB) buffer, and then labeled using 8-plex iTRAQ reagents (AB SCIEX) as manual described. The tryptic peptides from the Control, *ihfA*^L−^ (LihfA), *fadR*^H−^ (HfadR), and *fadR*^+^ (OfadR) strains were labeled with iTRAQ tags 113, 114, 115, and 116, respectively. After labeling, the samples were fractioned and then analyzed using a Q Exactive HF mass spectrometer (Thermo Scientific). The raw data obtained from mass spectrometry detection were deposited as ‘raw.7z’. Peptide identification and quantification were then conducted by Beijing Novogene Bioinformatics Technology Co., Ltd (China) using the Proteome Discoverer 2.2 software (Thermo Scientific), and the results were deposited as ‘search.7z’. The raw MS data ‘raw.7z’and the peptide identification and quantification data ‘search.7z’ are associated with the ProteomeXchange Consortium accession PXD017890.

### Transcriptomic analysis

Cells were harvested after 24 h of IPTG induction by quick centrifugation at 10,000 × *g* for 1 min and immediately frozen in liquid nitrogen. Total RNA was extracted using the RNAprep pure Cell/Bacteria Kit (Tiangen) following lysozyme treatment. RNA degradation and contamination were monitored on 1% agarose gels. RNA concentration was measured by Qubit^®^ RNA Assay Kit in Qubit^®^ 2.0 Flurometer (Life Technologies, CA, USA) and RNA integrity was assessed on a 2100 Bioanalyzer (Agilent Technologies, CA, USA). rRNA was removed using a Ribo-zero kit that left the mRNA. A total of 3 μg RNA was used as an input per sample. Sequencing libraries were generated using a NEBNext^®^UltraTM RNA Library Prep Kit for Illumina^®^ (NEB, USA) following the manufacturer’s instructions, and index codes were added to attribute sequences to each sample. Clustering of the index-coded samples was performed on a cBot Cluster Generation System using the TruSeq PE Cluster Kit v3-cBot-HS (Illumina) according to the manufacturer’s instructions. After cluster generation, the library preparations were sequenced on an Illumina Hiseq 4000 platform and paired-end reads were generated. The data were analyzed by Beijing Novogene Bioinformatics Technology Co., Ltd (China). Control, LihfA, HfadR, and OfadR represent the Control, *ihfA*^L−^, *fadR*^H−^ and *fadR*^+^ strains, respectively.

### Reporting summary

Further information on research design is available in the Nature Research Reporting Summary linked to this article.

## Supplementary information

Supplementary Information file

Supplementary Data 1-9

Description of Additional Supplementary Files

Reporting Summary

## Data Availability

Data supporting the findings of this work are available within the paper and its Supplementary Information files. A reporting summary for this article is available as a Supplementary Information file. The mass spectrometry-based proteome data generated in this study have been deposited in ProteomeXchange Consortium via the iProX partner repository under accession code PXD017890. The transcriptome data generated in this study have been deposited in Gene Expression Omnibus under accession code GSE146162. Nucleotide sequences of pCF, Sg-S, sgRNA0-biobrick, T7-T1, Trc-T1, and BAD-T1 have been deposited in NCBI Genbank under accession codes MZ567118, MZ567119, MZ567120, MZ567121, MZ567122, and MZ567123 respectively. GraphPad Prism 8 was used to plot data. Source data are provided with this paper.

## Source data

Source Data
