# Supplementary material for: Genome-scale target identification in Escherichia coli for high-titer production of free fatty acids
Source: Nat Commun. 2021 Aug 17;12:4976. doi: 10.1038/s41467-021-25243-w (PMC8371096; doi:10.1038/s41467-021-25243-w)
Supplement: Supplementary file 1 — Supplementary Information file [file 41467_2021_25243_MOESM1_ESM.pdf]

Genome-scale target identification in *Escherichia coli* for high-titer production of free fatty acids

Fang *et al.*

Supplementary Note 1. Construction of a functional CRISPRi system in *E. coli* BL21(DE3)

When CRISPRi technology is utilized in metabolic engineering, it is usually to first construct a functional CRISPRi system that can achieve high repression[1,2]. However, high-level dCas9 could cause severe inhibition of cell growth and abnormal expression of genes in a strain-specific manner[3,4]. Thus, it is extremely important to determine a superior dCas9 expression level. We constructed plasmids with dCas9 expressed under the control of four different promoters. The expression strengths of these promoters were in the order $P_{T7} > P_{T5} > P_{Trc} > P_{BAD}$[5]. With green fluorescent protein (GFP) as a characterization signal, fluorescence-based reporter plasmids were constructed. The plasmid Sg-*gfp*$^{H-}$ was constructed to express sgRNA *gfp*$^{H-}$, targeting the nontemplate strand of GFP at a position ~38 nucleotides downstream of the start codon[6]. We found that the OD_{600} of the G1 and G2 strains was greatly decreased compared with that of the G0 strain, while that of the G3 and G4 strains was hardly affected (Supplementary Fig. 1a). In addition, the strains with dCas9 regulated by the P_{Trc} promoter (g3 and G3) showed a 93% reduction in GFP fluorescence (Supplementary Fig. 1b). Thus, we selected dCas9 controlled by the P_{Trc} promoter to construct the CRISPRi system in BL21(DE3), achieving high repression efficiency without affecting cell growth.

Supplementary Note 2. Selection of candidate targets from differentially expressed genes for reverse engineering

The *norR*, *aroM*, *waaF*, *nrdE*, *npr*, *yihU*, *lpp*, *glpG*, *cirA*, *ftsQ*, *creC*, *yijO*, *sdhB*, *pal*, *rplW*, and *recN* genes were selected based on their significantly decreased abundance at the protein level in three FFAs-overproducing strains (fold change < 0.67, set a of Fig. 4b). We also selected 8 non-coding RNAs (ncRNAs) (namely, *tyrU*, *csrC*, *rnpB*, *ssrS*, *csrB*, *ssrA*, *ryfA*, and *sdsR*) with decreased abundance at the transcript level in two FFAs-overproducing strains (fold change < 0.5, set b of Fig. 4b). On the other hand, genes with increased abundance at the protein level (*folK*, *aidB*, *tam*, *yphF*, *deoD*, *ybhC*, *nnr*, *hpaG*, *rihC*, and *yajD*) (fold change > 1.5, set c of Fig. 4b) or at the transcript level (*hofP*, *nikB*, *1466*, *ulaE*, *torD*, *cheR*, *wcaF*) (fold change > 2, set d of Fig. 4b) in at least two FFAs-overproducing strains were all selected, except *tesA* (which was engineered in our starting strain) and *ileX* (encoding tRNA-Ile, which could not be regulated by our overexpression cassettes). Herein, we totally selected 41 targets, and the abundance of these genes at the protein and transcript levels are summarized in Fig. 4c.

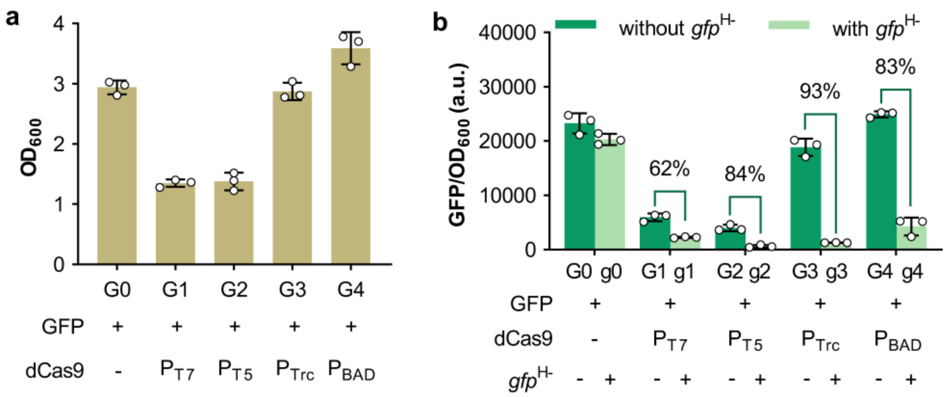

Supplementary Figure 1. Construction of a functional CRISPRi system in *E. coli* BL21(DE3). a Effect of dCas9 expression on cell growth. **b** Characterization of repression efficiency by GFP fluorescence. The repression efficiency is displayed by numbers above each bar, representing the decreased fluorescence of the strain co-expressing dCas9 and sgRNA *gfp*$^{H-}$ (light green bars) compared with that of the strain expressing only dCas9 (dark green bars). dCas9 was expressed under the control of four different promoters. *gfp* and *gfp*$^{H-}$ were expressed under the control of the P_{T7} promoter and the P_R promoter, respectively. Data are presented as mean ± SD (n = 3 biological replicates). Source data are provided as a Source Data file.

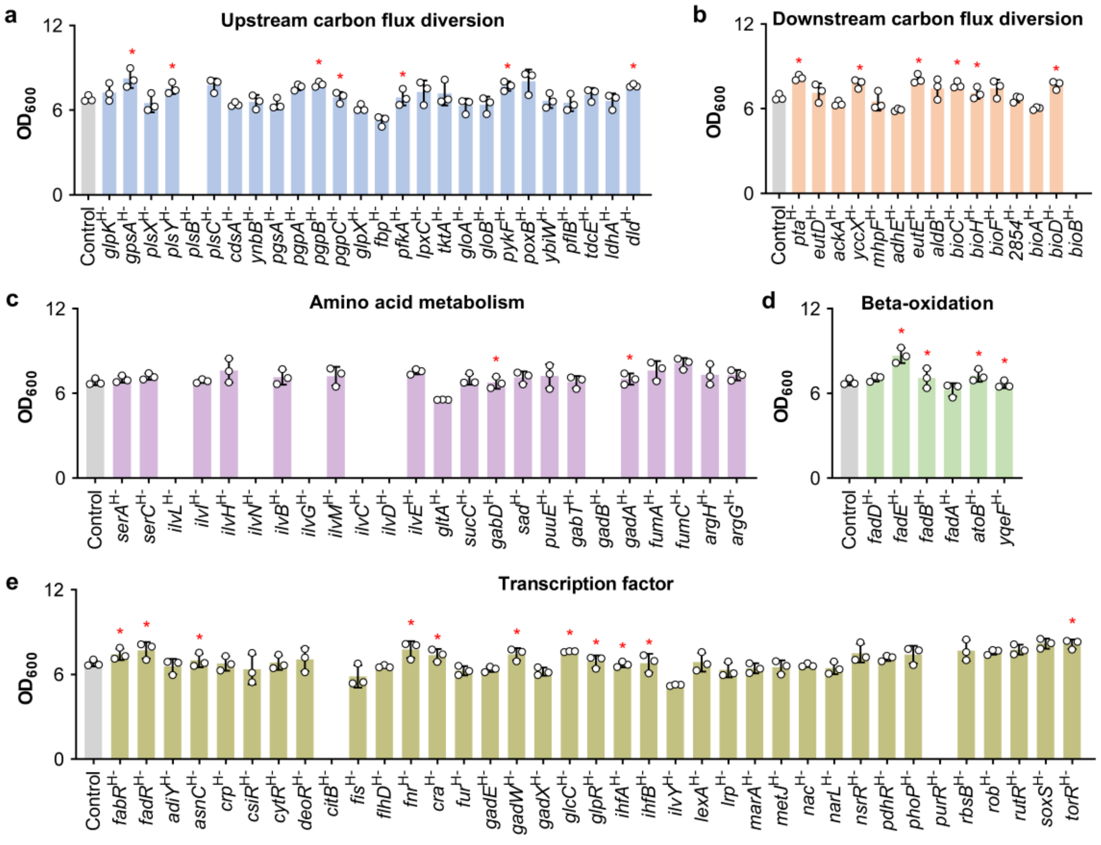

Supplementary Figure 2. Cell growth of strains with genetic perturbation by CRISPRi. All 108 genes in the upstream carbon flux diversion module (**a**), downstream carbon flux diversion module (**b**), amino acid metabolism module (**c**), beta-oxidation module (**d**), and transcription factor module (**e**) were repressed with high efficiency. Asterisk represents the strain in which the FFAs titer increased by over 20% compared with that in the Control strain (631 mg L^{-1}). Data are presented as mean ± SD (*n* = 3 biological replicates). Source data are provided as a Source Data file.

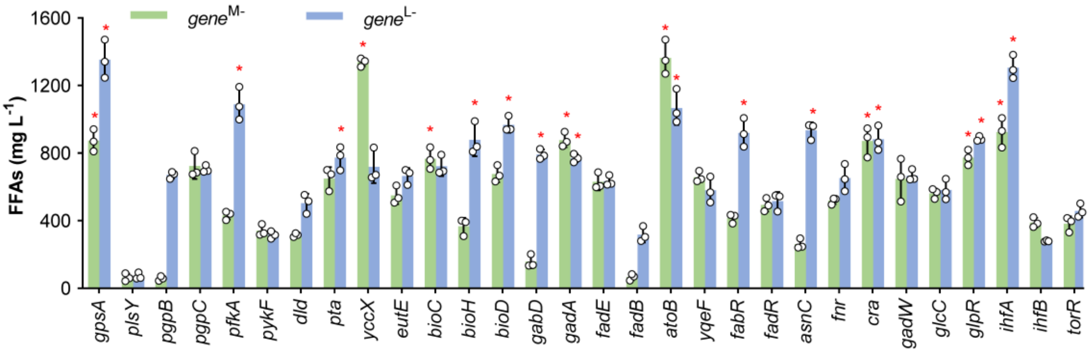

Supplementary Figure 3. Tuning of the repression of beneficial genes for FFAs production. The targeted genes were repressed with medium (*gene*$^{M-}$) or low (*gene*$^{L-}$) efficiency. Asterisk represents the strain in which the FFAs titer increased by over 20% compared with that in the Control strain (631 mg L^{-1}). The titers were obtained in batch cultivation of 30 g L^{-1} glycerol. Data are presented as mean ± SD (n = 3 biological replicates). Source data are provided as a Source Data file.

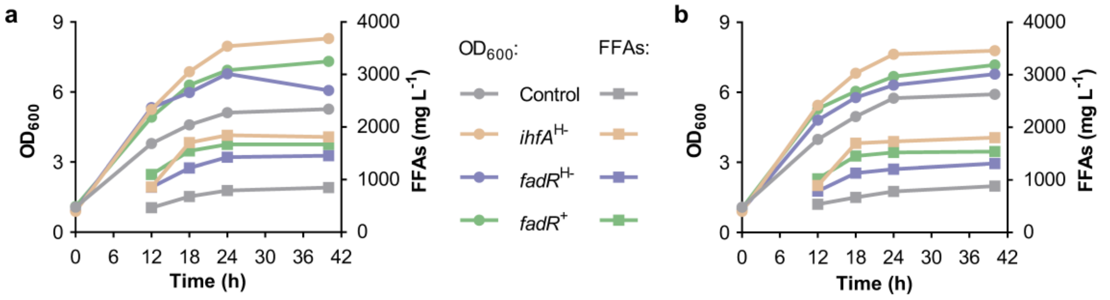

Supplementary Figure 4. Flask fermentation of strains for omics analyses. a Time courses of FFAs production and cell growth (OD_{600}) during the fermentation of strains sampled for omics analyses. **b** A duplicate fermentation exhibiting similar cell growth and FFAs production. The titers were obtained in batch cultivation of 30 g L^{-1} glycerol. Source data are provided as a Source Data file.

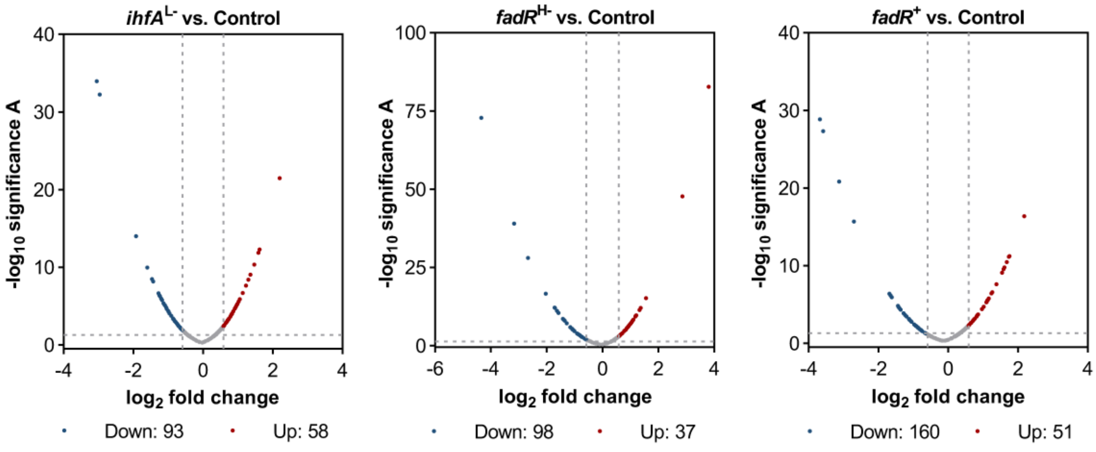

Supplementary Figure 5. Comparative proteomic analysis of the selected strains. Differentially expressed genes at the protein level (abs ($\log_2$ fold change) > 0.585 and significance A < 0.05) in *ihfA*$^{L-}$ vs. Control, *fadR*$^{H-}$ vs. Control, and *fadR*$^{+}$ vs. Control. The red dots indicate genes with increased abundance at the protein level, and the blue dots represent genes with decreased abundance at the protein level. significance A was used for detection of significant change of protein abundance, which was calculated by referring to a previous study of proteomic analysis[7]. Neither one-sided nor two-sided test was used, and no adjustments were made for multiple comparisons. Source data are provided as a Source Data file.

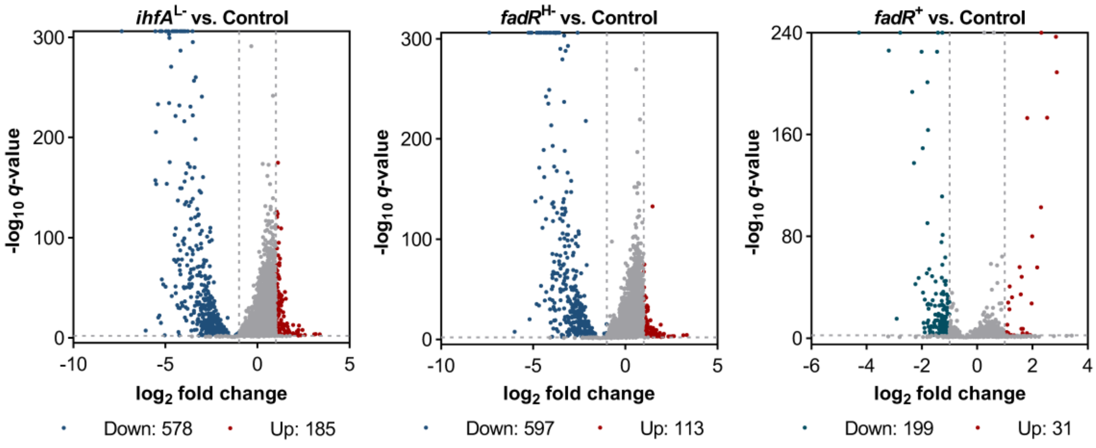

Supplementary Figure 6. Comparative transcriptomic analysis of the selected strains. Differentially expressed genes at the transcript level (abs ($\log_2$ fold change) > 1 and *q*-value < 0.005 (Benjamini–Hochberg method)) in *ihfA*$^{L-}$ vs. Control, *fadR*$^{H-}$ vs. Control, and *fadR*$^{+}$ vs. Control. The red dots indicate genes with increased abundance at the transcript level, and the blue dots represent genes with decreased abundance at the transcript level. *q*-value was calculated by using DEGseq 1.12.0 software[8]. Source data are provided as a Source Data file.

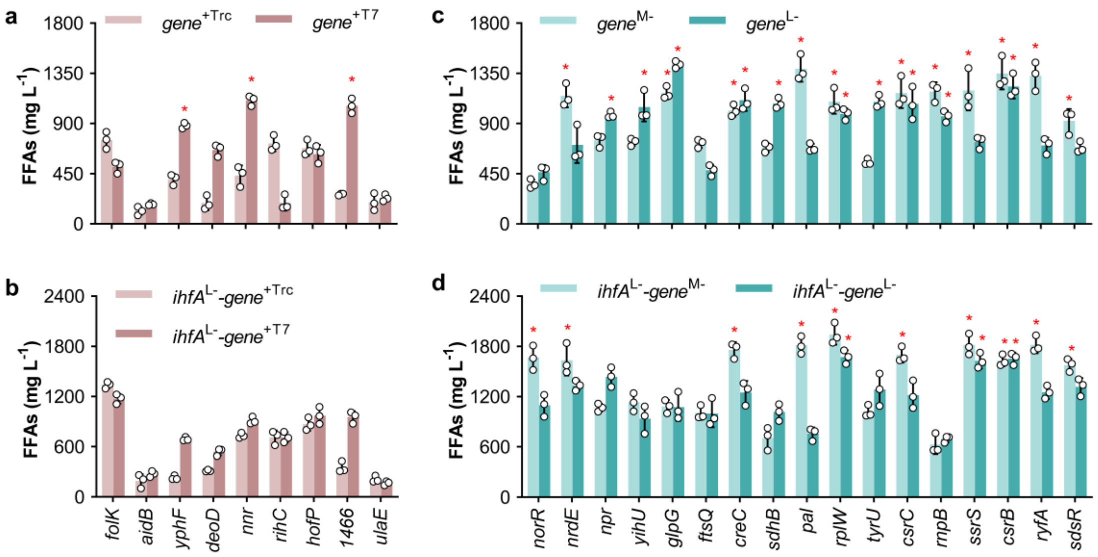

Supplementary Figure 7. Tuning of the expression of beneficial genes for FFAs production. FFAs production with genes overexpressed by the P_{Trc} or P_{T7} promoters in the CF strain (**a**) or in the *ihfA*$^{L-}$ strain (**b**). FFAs production with genes repressed with medium (*gene*$^{M-}$) or low (*gene*$^{L-}$) efficiency in the CF strain (**c**) or in the *ihfA*$^{L-}$ strain (**d**). Asterisk represents the strain in which the FFAs titer increased by over 20% compared with that in the reference strain Control or *ihfA*$^{L-}$. Red bar, overexpression; green bar, repression. The titers were obtained in batch cultivation of 30 g L^{-1} glycerol. Data are presented as mean ± SD (n = 3 biological replicates). Source data are provided as a Source Data file.

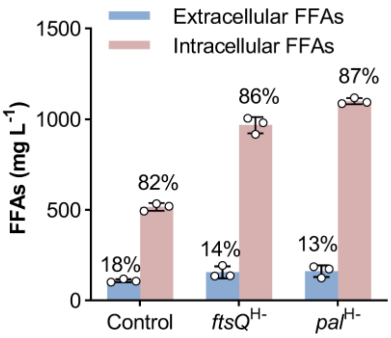

Supplementary Figure 8. Intracellular and extracellular FFAs produced by the Control, *ftsQ*$^{H-}$, and *pal*$^{H-}$ strains. The number above each bar represents the percentage of total FFAs. The titers were obtained in batch cultivation of 30 g L^{-1} glycerol. Data are presented as mean ± SD (n = 3 biological replicates). Source data are provided as a Source Data file.

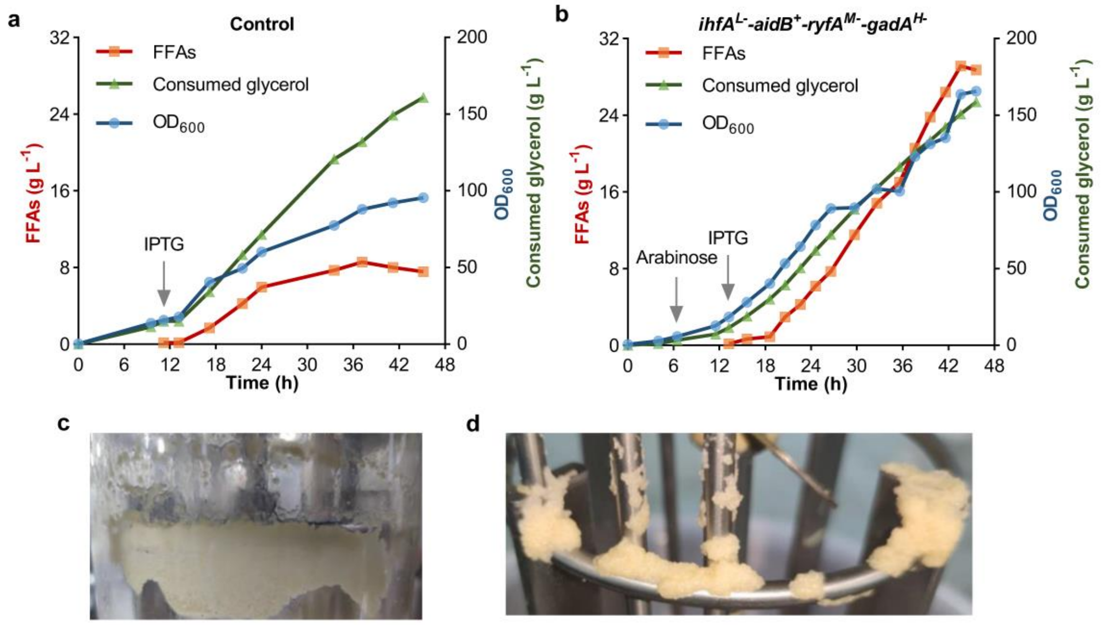

Supplementary Figure 9. Fed-batch fermentation of the Control and *ihfA*$^{L-}$-*aidB*$^{+}$-*ryfA*$^{M-}$-*gadA*$^{H-}$ strains in 5 L bioreactors. Time courses of cell growth, glycerol consumption, and FFAs production of the Control strain (**a**) and *ihfA*$^{L-}$-*aidB*$^{+}$-*ryfA*$^{M-}$-*gadA*$^{H-}$ strain (Batch 2) (**b**) during fed-batch fermentation. When cells were cultivated for 37.17 h, the Control strain produced 8.6 g L^{-1} FFAs with productivity of 0.231 g L^{-1} h^{-1}. When cells were cultivated for 43.60 h, the engineered strain *ihfA*$^{L-}$-*aidB*$^{+}$-*ryfA*$^{M-}$-*gadA*$^{H-}$ produced 29.1 g L^{-1} FFAs with productivity of 0.667 g L^{-1} h^{-1}. Floating dead cells or fatty acid particles stuck to the fermenter inner wall (**c**) and the sensors (**d**) during the fermentation of the *ihfA*$^{L-}$-*aidB*$^{+}$-*ryfA*$^{M-}$-*gadA*$^{H-}$ strain. Source data underlying Supplementary Figure 9a and 9b are provided as a Source Data file.

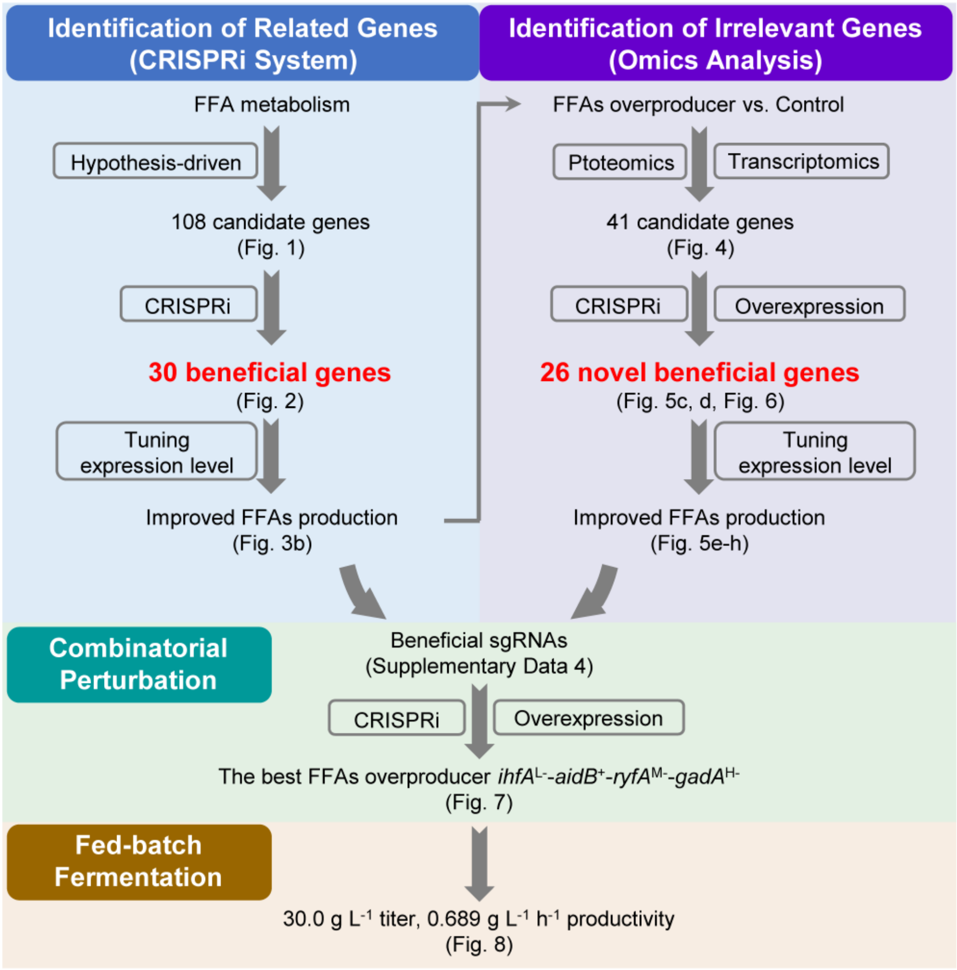

Supplementary Figure 10. Workflow for identification of beneficial genes and engineering *E. coli* for high-titer production of FFAs in our work.

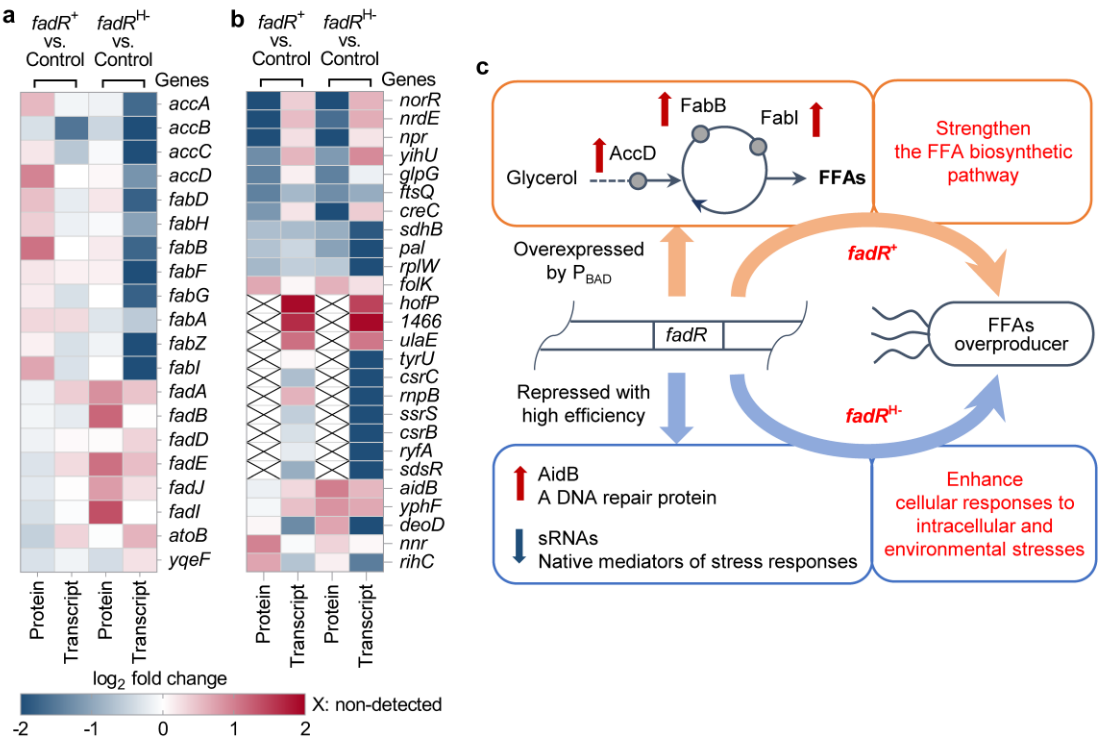

Supplementary Figure 11. Analysis of the mechanism of FFAs overproduction in the *fadR*$^{+}$ and *fadR*$^{H-}$ strains. The protein and transcript abundances of genes in the FFA biosynthetic and degradation pathways (**a**) and of the beneficial genes identified from omics analyses (**b**) in the *fadR*$^{+}$ and *fadR*$^{H-}$ strains compared with the Control strain. **c** Possible mechanism of FFAs overproduction in the *fadR*$^{+}$ and *fadR*$^{H-}$ strains. Dark red arrow, with increased abundance at the protein level; dark blue arrow, with decreased abundance at the transcript level. Source data underlying Supplementary Figure 11a and 11b are provided as a Source Data file.

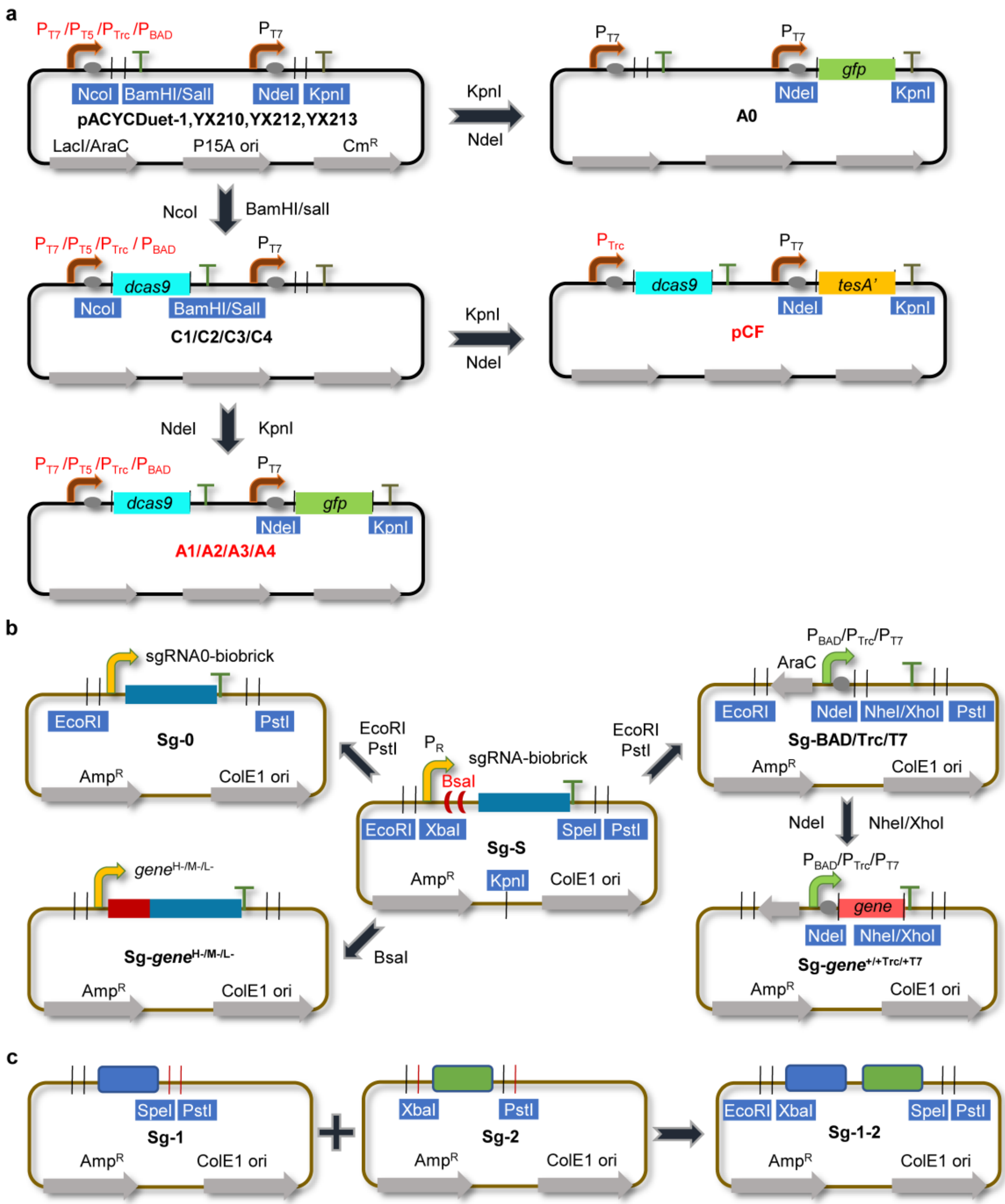

Supplementary Figure 12. Construction of plasmids. a Cloning *dcas9* into the NcoI and BamHI/SalI sites of pACYCDute-1, YX210, YX212, and YX213 resulted in plasmids C1-C4, respectively. Then, subcloning *gfp* into the NdeI and KpnI sites of pACYCDute-1 and plasmids C1-C4 resulted in plasmids A0-A4, respectively. Alternatively, subcloning *tesA'* into the NdeI and KpnI sites of plasmid C3 resulted in plasmid pCF. **b** Plasmid Sg-S was constructed via ligation of the synthesized fragments sgRNA-biobrick-ColE1 and Amp-mBsaI after digestion by EcoRI and KpnI. Plasmid Sg-0 was constructed by inserting the synthesized fragments of sgRNA0-biobrick into plasmid Sg-S between the EcoRI and PstI sites. Cloning the

20-bp targeting sequence of sgRNA *gfp*$^{H-}$ or other sgRNAs into the two BsaI sites of plasmid Sg-S resulted in various sgRNA expression plasmids, Sg-*gene*$^{H/M/L-}$. Fragments BAD-T1, Trc-T1, and T7-T1 were synthesized, digested by EcoRI and PstI, and then ligated into Sg-S, resulting in plasmids Sg-BAD, Sg-Trc, and Sg-T7, respectively. Genes that needed to be upregulated were amplified from *E. coli* genomic DNA and inserted into Sg-BAD, Sg-Trc, and Sg-T7 between the NdeI and NheI/XhoI sites, resulting in plasmids Sg-*gene*$^{+}$, Sg-*gene*$^{+Trc}$, and Sg-*gene*$^{+T7}$, respectively. **c** Plasmid Sg-1-2 was constructed via ligation of the sgRNA fragment from Sg-2 digested with XbaI and PstI into Sg-1 digested with SpeI and PstI. Sg-1, plasmid expressing the first sgRNA; Sg-2, plasmid expressing the second sgRNA; Sg-1-2, plasmid expressing two sgRNAs.

Supplementary Table 1. Comparison of FFAs production by microbial fermentation in bioreactors.

**Microorganism**	**Feedstock**	**Titer (g L^{-1})**	**Productivity (g L^{-1} h^{-1})**	**Cultivation time (h)**	**Cultivation mode**	**Reference**
Escherichia coli	Glucose	8.6	0.124	~70	Fed-batch	Xu *et al.*[9]
Escherichia coli	Glycerol	15.67	~0.348	~45	Fed-batch	Wu *et al.*[10]
Escherichia coli	Glucose	21.5	0.5	49.2	Fed-batch	Xiao *et al.*[11]
Escherichia coli	Glycerol	30.0	0.689	43.55	Fed-batch	This study
Saccharomyces cerevisiae	Glucose	10.4	~0.087	~120	Fed-batch	Zhou *et al.*[12]
Saccharomyces cerevisiae	Glucose	33.4	<0.145	>230	Fed-batch	Yu *et al.*[13]
Yarrowia lipolytica	Glucose	9.67	<0.088	>110	Fed-batch	Xu *et al.*[14]
Yarrowia lipolytica	Glucose	10.4	~0.074	~140	Batch	Ledesma-Amaro *et al.*[15]
Rhodococcus opacus	Glucose	50.2	0.478	104.7	Fed-batch	Kim *et al.*[16]

Supplementary Table 2. FFAs production in *E. coli* using glycerol or glucose as feedstock.

**Feedstock**	**Titer (g L^{-1})**	**Productivity (g L^{-1} h^{-1})**	**Cultivation time (h)**	**Cultivation mode**	**Reference**
Glucose	2.97	0.041	72	Shake flask	Li *et al.*[17]
Glucose	3.86	0.088	44	Fed-batch	Xu *et al.*[18]
Glucose	4.7	0.098	>48	Shake flask	Wu *et al.*[19]
Glucose	8.6	0.124	~70	Fed-batch	Xu *et al.*[9]
Glucose	21.5	0.5	49.2	Fed-batch	Xiao *et al.*[11]
Glycerol	2.5	~0.114	~22	Fed-batch	Lu *et al.*[20]
Glycerol	3.43	0.048	72	Shake flask	Li *et al.*[17]
Glycerol	4.82	0.067	72	Shake flask	Wu *et al.*[21]
Glycerol	15.67	0.348	~45	Fed-batch	Wu *et al.*[10]
Glycerol	30.0	0.689	43.55	Fed-batch	This study

Supplementary references

1. Wu, J., Du, G., Chen, J. & Zhou, J. Enhancing flavonoid production by systematically tuning the central metabolic pathways based on a CRISPR interference system in *Escherichia coli*. *Sci. Rep.* **5**, 13477 (2015).
2. Tian, T., Kang, J. W., Kang, A. & Lee, T. S. Redirecting metabolic flux via combinatorial multiplex CRISPRi-mediated repression for isopentenol production in *Escherichia coli*. *ACS Synth. Biol.* **8**, 391-402 (2019).
3. Cho, S. et al. High-Level dCas9 Expression induces abnormal cell morphology in *Escherichia coli*. *ACS Synth. Biol.* **7**, 1085-1094 (2018).
4. Cui, L. et al. A CRISPRi screen in *E. coli* reveals sequence-specific toxicity of dCas9. *Nat. Commun.* **9**, 1912 (2018).
5. Cao, Y. X. et al. Biosynthesis of odd-chain fatty alcohols in *Escherichia coli*. *Metab. Eng.* **29**, 113-123 (2015).
6. Cao, Y., Li, X., Li, F. & Song, H. CRISPRi-sRNA: transcriptional-translational regulation of extracellular electron transfer in *Shewanella oneidensis*. *ACS Synth. Biol.* **6**, 1679-1690 (2017).
7. Cox, J. & Mann, M. MaxQuant enables high peptide identification rates, individualized p.p.b.-range mass accuracies and proteome-wide protein quantification. *Nat. Biotechnol.* **26**, 1367-1372 (2008).
8. Wang, L., Feng, Z., Wang, X., Wang, X. & Zhang, X. DEGseq: an R package for identifying differentially expressed genes from RNA-seq data. *Bioinformatics* **26**, 136-138 (2010).
9. Xu, P. et al. Modular optimization of multi-gene pathways for fatty acids production in *E. coli*. *Nat. Commun.* **4**, 1409 (2013).
10. Wu, J. et al. Construction of artificial micro-aerobic metabolism for energy- and carbon-efficient synthesis of medium chain fatty acids in *Escherichia coli*. *Metab. Eng.* **53**, 1-13 (2019).
11. Xiao, Y., Bowen, C. H., Liu, D. & Zhang, F. Exploiting nongenetic cell-to-cell variation for enhanced biosynthesis. *Nat. Chem. Biol.* **12**, 339-344 (2016).
12. Zhou, Y. J. et al. Production of fatty acid-derived oleochemicals and biofuels by synthetic yeast cell factories. *Nat. Commun.* **7**, 11709 (2016).
13. Yu, T. et al. Reprogramming yeast metabolism from alcoholic fermentation to lipogenesis. *Cell* **174**, 1549-1558 e1514 (2018).
14. Xu, P., Qiao, K., Ahn, W. S. & Stephanopoulos, G. Engineering *Yarrowia lipolytica* as a platform for synthesis of drop-in transportation fuels and oleochemicals. *Proc. Natl. Acad. Sci. U. S. A.* **113**, 10848-10853 (2016).
15. Ledesma-Amaro, R., Dulermo, R., Niehus, X. & Nicaud, J. M. Combining metabolic engineering and process optimization to improve production and secretion of fatty acids. *Metab. Eng.* **38**, 38-46 (2016).
16. Kim, H. M., Chae, T. U., Choi, S. Y., Kim, W. J. & Lee, S. Y. Engineering of an oleaginous bacterium for the production of fatty acids and fuels. *Nat. Chem. Biol.* **15**, 721-729 (2019).
17. Li, W., Wu, H., Li, M. & San, K. Y. Effect of NADPH availability on free fatty acid production in *Escherichia coli*. *Biotechnol. Bioeng.* **115**, 444-452 (2018).

18. Xu, P., Li, L., Zhang, F., Stephanopoulos, G. & Koffas, M. Improving fatty acids production by engineering dynamic pathway regulation and metabolic control. *Proc. Natl. Acad. Sci. U. S. A.* **111**, 11299-11304 (2014).
19. Wu, J. et al. Improving metabolic efficiency of the reverse beta-oxidation cycle by balancing redox cofactor requirement. *Metab. Eng.* **44**, 313-324 (2017).
20. Lu, X., Vora, H. & Khosla, C. Overproduction of free fatty acids in *E. coli*: implications for biodiesel production. *Metab. Eng.* **10**, 333-339 (2008).
21. Wu, H., Karanjikar, M. & San, K. Y. Metabolic engineering of *Escherichia coli* for efficient free fatty acid production from glycerol. *Metab. Eng.* **25**, 82-91 (2014).
