# Supplementary material for: Genome-scale target identification in Escherichia coli for high-titer production of free fatty acids
Source: Nat Commun. 2021 Aug 17;12:4976. doi: 10.1038/s41467-021-25243-w (PMC8371096; doi:10.1038/s41467-021-25243-w)
Supplement: Supplementary file 3 — Description of Additional Supplementary Files [file 41467_2021_25243_MOESM3_ESM.pdf]

Description of Additional Supplementary Files

File Name: Supplementary Data 1

Description: List of genes targeted for FFAs metabolic engineering in this study.

File Name: Supplementary Data 2

Description: Fold change in the abundance of the protein or transcript.

File Name: Supplementary Data 3

Description: Genes in the functional network and the corresponding FFAs ratio.

File Name: Supplementary Data 4

Description: List of beneficial sgRNAs.

File Name: Supplementary Data 5

Description: Strains used in this study.

File Name: Supplementary Data 6

Description: Plasmids used in this study.

File Name: Supplementary Data 7

Description: Primers used to construct plasmids Sg-$\textit{gene}^{H/M/L-}$.

File Name: Supplementary Data 8

Description: Primers used to amplify gene from *E. coli* genome.

File Name: Supplementary Data 9

Description: DNA sequences used in this study.
