# Supplementary material for: Genome-scale target identification in Escherichia coli for high-titer production of free fatty acids
Source: Nat Commun. 2021 Aug 17;12:4976. doi: 10.1038/s41467-021-25243-w (PMC8371096; doi:10.1038/s41467-021-25243-w)
Supplement: Supplementary file 4 — Reporting Summary [file 41467_2021_25243_MOESM4_ESM.pdf]

nature research

Corresponding author(s): Hao Song, Yingxiu Cao

Last updated by author(s): Jul 19, 2021

Reporting Summary

Nature Research wishes to improve the reproducibility of the work that we publish. This form provides structure for consistency and transparency in reporting. For further information on Nature Research policies, see our Editorial Policies and the Editorial Policy Checklist.

Statistics

For all statistical analyses, confirm that the following items are present in the figure legend, table legend, main text, or Methods section.

n/a	Confirmed	
☐	☒	The exact sample size (*n*) for each experimental group/condition, given as a discrete number and unit of measurement
☐	☒	A statement on whether measurements were taken from distinct samples or whether the same sample was measured repeatedly
☐	☒	The statistical test(s) used AND whether they are one- or two-sided<br>*Only common tests should be described solely by name; describe more complex techniques in the Methods section.*
☒	☐	A description of all covariates tested
☒	☐	A description of any assumptions or corrections, such as tests of normality and adjustment for multiple comparisons
☐	☒	A full description of the statistical parameters including central tendency (e.g. means) or other basic estimates (e.g. regression coefficient) AND variation (e.g. standard deviation) or associated estimates of uncertainty (e.g. confidence intervals)
☐	☒	For null hypothesis testing, the test statistic (e.g. *F*, *t*, *r*) with confidence intervals, effect sizes, degrees of freedom and *P* value noted<br>*Give P values as exact values whenever suitable.*
☒	☐	For Bayesian analysis, information on the choice of priors and Markov chain Monte Carlo settings
☒	☐	For hierarchical and complex designs, identification of the appropriate level for tests and full reporting of outcomes
☒	☐	Estimates of effect sizes (e.g. Cohen's *d*, Pearson's *r*), indicating how they were calculated

Our web collection on statistics for biologists contains articles on many of the points above.

Software and code

Policy information about availability of computer code

Data collection	The titer of FFAs and glycerol were quantified using the Chromeleon 7.1 and Empower3 softwares, respectively. Fluorescence intensity of GFP was detected by the SoftMax Pro 5.0.1 software. For proteomics, the Q Exactive HF mass spectrometer (Thermo Scientific) with Tune 2.11 was used to separate the peptides and generate the raw data. For transcriptomics, illumina Hiseq 4000 platform with Casava 1.8 was used to sequence the transcript and generate paired-end reads.
Data analysis	Proteome Discoverer 2.2 (Thermo Scientific) was used for peptide identification and quantification. DEGseq 1.12.0 was used for transcriptomic analysis. GraphPad Prism 8.2.1 was used to plot data.

For manuscripts utilizing custom algorithms or software that are central to the research but not yet described in published literature, software must be made available to editors and reviewers. We strongly encourage code deposition in a community repository (e.g. GitHub). See the Nature Research guidelines for submitting code & software for further information.

Data

Policy information about availability of data

All manuscripts must include a data availability statement. This statement should provide the following information, where applicable:

- Accession codes, unique identifiers, or web links for publicly available datasets
- A list of figures that have associated raw data
- A description of any restrictions on data availability

Data supporting the findings of this work are available within the paper and its Supplementary Information files. A reporting summary for this Article is available as a Supplementary Information file. All the datasets generated and analyzed in the paper are available from the corresponding author upon request. The mass spectrometry-based proteome data generated in this study have been deposited in ProteomeXchange Consortium via the iProX partner repository under accession code PXD017890 (http://proteomecentral.proteomexchange.org/cgi/GetDataset?ID=PXD017890). The transcriptome data generated in this study have been

deposited in Gene Expression Omnibus under accession code GSE146162 (https://www.ncbi.nlm.nih.gov/geo/query/acc.cgi?acc=GSE146162). Nucleotide sequences of pCF, Sg-S, sgRNA0-biobrick, T7-T1, Trc-T1, and BAD-T1 have been deposited in NCBI Genbank (https://www.ncbi.nlm.nih.gov/genbank/) under accession codes MZ567118 to MZ567123, respectively. The source data underlying Figs. 2a-e, 3b, 4a-c, 5c-h, 6, 7a-b, and 8a, Supplementary Figs. 1a-b, 2a-e, 3, 4a-b, 5, 6, 7a-d, 8, 9a-b, and 11a-b are provided as a Source Data file.

Field-specific reporting

Please select the one below that is the best fit for your research. If you are not sure, read the appropriate sections before making your selection.

☒ Life sciences ☐ Behavioural & social sciences ☐ Ecological, evolutionary & environmental sciences

For a reference copy of the document with all sections, see nature.com/documents/nr-reporting-summary-flat.pdf

Life sciences study design

All studies must disclose on these points even when the disclosure is negative.

Sample size	Sample size was determined by multiple pilot experiments as described in the manuscript.
Data exclusions	No data were excluded from the analyses.
Replication	All attempts at replication were successful. Tube cultivations were performed in three biological replicates. Flask cultivations were performed in two biological replicates. For fed-batch fermentation, the Control strain and the engineered FFAs overproducing strain were performed in one and two biological replicates, respectively. Proteomic and transcriptomic analyses were performed in one replicate, which could successfully provide effective targets for strain engineering.
Randomization	Colonies are picked randomly from the agar plates.
Blinding	Blinding was not possible as we need to know the performance of each engineered strain. The samples of the bacterial cultures were random samplings. None of our data is involving animals or human research participants.

Reporting for specific materials, systems and methods

We require information from authors about some types of materials, experimental systems and methods used in many studies. Here, indicate whether each material, system or method listed is relevant to your study. If you are not sure if a list item applies to your research, read the appropriate section before selecting a response.

Materials & experimental systems

n/a	Involved in the study	
☒	☐	Antibodies
☒	☐	Eukaryotic cell lines
☒	☐	Palaeontology and archaeology
☒	☐	Animals and other organisms
☒	☐	Human research participants
☒	☐	Clinical data
☒	☐	Dual use research of concern

Methods

n/a	Involved in the study	
☒	☐	ChIP-seq
☒	☐	Flow cytometry
☒	☐	MRI-based neuroimaging
